# Antagonizing NRG1-ERBB4 signaling pathway with spironolactone for the treatment of schizophrenia: results of a randomized controlled drug repositioning clinical trial

**DOI:** 10.1038/s43856-026-01714-3

**Published:** 2026-06-16

**Authors:** Alkomiet Hasan, Stefan Leucht, Astrid Roeh, Berthold Langguth, Maximilian Hansbauer, Tatiana Oviedo-Salcedo, Sophie K. Greiner, Irina Papazova, Lisa Löhrs, Isabel Maurus, Wolfgang Strube, Moritz J. Rossner, Michael C. Wehr, Ingrid Bauer, Stephan Heres, Peter M. Kreuzer, Stephanie Zimmermann, Thomas Schneider-Axmann, Thomas Görlitz, Anja Baumgartner, Susanne Karch, Silvia Egert-Schwender, Christiane Blankenstein, Philipp Rothe, Elias Wagner, Zahra Aminifarsani, Bryan Downie, Claudia Leucht, Peter Falkai

**Affiliations:** 1 Department of Psychiatry, Psychotherapy and Psychosomatics, Medical Faculty, University of Augsburg, Augsburg, Germany; 2 DZPG (German Center for Mental Health), Partner Site München/Augsburg, Munich, Germany; 3 Department of Psychiatry and Psychotherapy, University Hospital, Technical University of Munich, Munich, Germany; 4 Department of Psychiatry and Psychotherapy, University of Regensburg, Bezirksklinikum, Regensburg, Germany; 5 Department of Psychiatry and Psychotherapy, University Hospital, Ludwig-Maximilians University, Munich, Germany; 6 Systasy Bioscience GmbH, Martinsried, Germany; 7 Department of Forensic Psychiatry and Psychotherapy, University of Regensburg, Bezirksklinikum, Regensburg, Germany; 8 Münchner Studienzentrum, TUM School of Medicine and Health, Munich, Germany; 9 Department of Forensic Psychiatry and Psychotherapy, Ulm University, Ulm, Germany; 10 Evidence-based Psychiatry and Psychotherapy, Faculty of Medicine, University of Augsburg, Augsburg, Germany; 11 Bayesian Imaging and Spatial Statistics Group, Institute for Statistics, Ludwig-Maximilians University, Munich, Germany; 12 Node Biosciences,Inc., Columbus, OH USA; 13 Max-Planck-Institute of Psychiatry, München, Germany

**Keywords:** Schizophrenia, Clinical pharmacology

## Abstract

**Background:**

The (NRG1)-*ERBB4* signaling pathway has been identified to be pathophysiologically meaningful for cognitive impairments in schizophrenia. The in the 1960s approved mineralocorticoid antagonist spironolactone has been shown to be effective to modulate (NRG1)-ERBB4 signaling in preclinical and animal models. Thus, a proof-of-concept drug repurposing trial in patients with schizophrenia was justified.

**Methods:**

We conducted a multi-site proof-of-concept randomized controlled trial. 90 patients with schizophrenia were randomized to a three-week trial of two different spironolactone dosages (100 mg or 200 mg) or placebo. The primary endpoint was predefined as change in working memory performance after three weeks of treatment. A naturalistic follow-up was performed nine weeks after the end of intervention. The trial was registered at the WHO International Clinical Trials Registry platform (http://apps.who.int/trialsearch/Trial2.aspx?TrialID=EUCTR2014-001968-35-DE).

**Results:**

Despite large numerical improvements in working memory functions, particularly in the spironolactone 200 mg group, the pre-specified analyses do not demonstrate significant superiority of either intervention over placebo. However, post-hoc sensitivity analyses suggest a significant advantage of spironolactone for the primary endpoint. Safety measures show that both interventions are well tolerated.

**Conclusions:**

This finding suggests a potential positive effect of spironolactone on working memory in people with schizophrenia and the good safety outcomes may justify further trials with longer intervention periods or higher spironolactone dosages.

## Plain language summary

Individuals diagnosed with schizophrenia frequently experience cognitive deficits, including challenges related to everyday memory and attention. We evaluated whether spironolactone, a drug available since the 1960s and today used to treat heart failure is effective in treating cognitive impairment in people with schizophrenia. For that, we conducted a placebo-controlled clinical trial where participants received either 100 mg or 200 mg spironolactone or placebo for three weeks. Before and after this intervention, we tested cognition. We were able to show that our intervention with spironolactone has the potential to improve cognition in people with schizophrenia. Further trials are needed before this intervention can be offered as part of the clinical routine.

## Abstract (web summary)

Hasan, Leucht et al. undertook a drug repurposing trial evaluating the mineralocorticoid antagonist spironolactone as a treatment for cognitive impairment in people with schizophrenia. The primary analysis yielded negative results, but subsequent sensitivity analyses suggest this approach could potentially be a treatment option for schizophrenia.

## Introduction

Cognitive impairments are a core symptom domain of schizophrenia and one of the most significant predictors of social and vocational disability as discussed for long time^1^. Despite many different approaches, no effective pharmacological treatment options could yet be introduced addressing cognitive impairments in schizophrenia (CIAS)^2,3^. Antipsychotics with an established efficacy to treat acute psychosis^4^ and to prevent relapses^5^, have not been shown to be relevantly effective for cognitive symptoms and are not procognitive drugs^6,7^. Other pharmacological options, such as alpha-7 nicotinic acetylcholine receptor agonists^8^, minocycline^9^ or anti-dementia drugs^10^ were either not effective or not clinical meaningful for this indication. Novel antipsychotic concepts (e.g., drugs that combine sigma-2 antagonism and 5-HT_2A_ antagonism^11^) showed some effects on cognitive impairments in schizophrenia patients without a clear link to the neurobiology^11^. Moreover, promising new compounds with alternative modes of actions (e.g., glutamatergic modulation) failed before market launch^12–14^. Psychotherapeutic treatments^15^ applied in addition to antipsychotic medication were shown to improve cognitive impairments to a limited extent but were not capable to change clinical care so far as they require a relatively high degree of adherence.

This highlights the urgent need to develop novel treatment options for cognitive impairments in schizophrenia that are rooted in the disorder’s pathophysiology. Despite interesting new developments (e.g., trace amine-associated receptor agonists, antagonists at the sigma receptor) many pharmaceutical companies are withdrawing from the field of schizophrenia research. Some years ago, the National Institute of Mental Health (NIMH) implemented the concept of drug repurposing based on pathophysiological considerations in academia settings as one possibility to overcome the innovation blockade in psychopharmacological research^16,17^.

As outlined elsewhere^18,19^, the (NRG1)-ERBB4 signaling pathway has been identified to be pathophysiologically meaningful for schizophrenia on a multi-level consideration including an involvement of this pathway in working memory functions, alterations of neuronal inhibitory functions, the excitation-inhibition-balance, and impaired synaptic functioning^20–27^. More recently, ERBB4 has been identified also among the few druggable and credible GWAS risk genes for bipolar disorder^28^. Intriguingly, alterations in that signaling pathway have been related to a potentially reversible hyperphosphorylation of ERBB4^29,30^, opening the possibility of targeting this pathway with pharmacological compounds. Members of our group were the first to apply a drug repurposing strategy to identify potential pharmacological compounds that may normalize impaired signaling in the (NRG1)-ERBB4 pathway^18,19^. In short, the NIH-NCC compound library of approved drugs for chemical modulators was screened via a cell-based assay to identify compounds that may modulate NRG1-ERBB4 signaling^18,19^. This approach detected the mineralocorticoid antagonist spironolactone (approved in the 1960s for the treatment of heart failure) as significant inhibitor of *ERBB4* activity resulting in reduced phosphorylation levels of *ERBB4* both in vitro in human heterologous T-47D cells and in vivo in NRG1-transgenic mice, and moreover lead to an enhanced inhibitory neurotransmission following spironolactone admission in organotypic slice cultures^18,19^. Next, the behavioral impact of our newly detected preclinical approach was tested by treating NRG1 transgenic mice with spironolactone (i.e., daily injections for 3 weeks) resulting in an improvement in the preclinical model domains of positive symptoms (locomotor activity), sensory gating and working memory functions^18,19^. Moreover, spironolactone alleviated reversal learning in a TCF4 gain-of-function mouse model of cognitive dysfunction of relevance for schizophrenia^31^. Based on these promising preclinical findings, we performed a clinical study applying spironolactone to patients with schizophrenia^18^. The “Add-on spironolactone for the treatment of schizophrenia” (SPIRO-TREAT) trial was designed to test the efficacy of two different dosages of spironolactone for the treatment of cognitive impairments in schizophrenia according to the NIMH principles of translating biological and preclinical findings to investigator-initiated trial in academia.

Although working memory performance shows a relevant numerical improvement after the intervention, particularly with spironolactone 200 mg, planned analyses do not show a significant advantage for the study drugs. However, post-hoc sensitivity analyses reveal that spironolactone is significantly superior to placebo for the primary endpoint indicating a potential new option to treat cognitive impairments in schizophrenia. Confirmatory multi-centric RCTs are needed to replicate our findings.

## Methods

### Study design and participants

The trial concept has been previously published^18^ and is summarized in the following paragraphs. SPIRO-TREAT was designed as a multicenter (LMU Munich, TU Munich, University of Regensburg, all university hospitals), double-blind, three-group, randomized controlled trial. Inclusion criteria were^18^: (1) in- and outpatients (men and women) aged between 18 and 65 years with a primary diagnosis of schizophrenia according to ICD-10 confirmed by the Mini-International Neuropsychiatric Interview, (2) participants are able to sign informed consent, (3) participants must receive a stable antipsychotic treatment for at least one week, (4) participants must not be treated with more than two antipsychotics; (5) participants must have a PANSS total score ≤75, (6) participants must have a duration of illness of at least six months, (7) female participants must have a negative pregnancy test (serum) at baseline and must use a method of contraception that is medically approved by the health authority („Recommendations related to contraception and pregnancy testing in clinical trials (CTFG 2014). Exclusion criteria were^18^: (1) incapacity to give informed consent, (2) suicidality or endangerment of others, (3) severe somatic or neurological comorbidities, (4) history or assumption of relevant non-compliance that interferes with the ability to participate in a clinical trial, (5) current antipsychotic treatment with clozapine or an antipsychotic with exclusive renal elimination (e.g., amisulpride), (6) planned initiation of a treatment with an antidepressant or mood stabilizer during the intervention period (a prior treatment with an non-renal eliminated antidepressant or a mood-stabilizer other than lithium is permitted), (7) diagnoses of drug dependency other than tobacco or caffeine within the last 6 months prior to inclusion, (8) history of seizures, (9) documented intolerance to a treatment with spironolactone or placebo capsules, (10) acute kidney failure or anuria, or severe kidney insufficiency (creatinine clearance <30 ml/min per 1.73 m^2^ or serum creatinine > 1.8 mg/dl), (11) Clinically relevant hyperkalemia or hyponatremia, (12) clinically relevant hypotension (RR < 100/80 mmHg), (13) simultaneous use of potassium-sparing diuretics, ACE inhibitors, ATIIantagonists, non-steroidal anti-inflammatory drugs (NSAID), thiazide, diuretics, carbenoxolone, digoxin or neomycin, (14) coercive treatment, (15) treatment-resistant or treatment-naïve schizophrenia, (16) insufficient understanding of German language, (17) pregnancy, (18) Absent safe and approved methods of contraception.

More information regarding trial conduction is presented elsewhere^18^ and in the supplement (supplementary Notes 1 to 7; supplementary Table 1).

#### Trial registration

Prior to the inclusion of the first patient, the study was registered in the EU Clinical Trials Register (https://www.clinicaltrialsregister.eu/ctr-search/search?query=eudract_number:2014-001968-35) and the International Clinical Trials Registry Platform (ICTRP, http://apps.who.int/trialsearch/Trial2.aspx?TrialID=EUCTR2014-001968-35-DE).

### Ethics statement

The study was approved by the German Federal Institute for Drugs and Medical Devices (23.02.2015) and the respective ethics committees (11.03.2015 and 15.10.2025; Ethikkommission der Medizinischen Fakultät der LMU München (32-15 fed; Ethikkommission der Fakultät für Medizin der TU München (32-15 Am; Universität Regensburg Ethikkommission (17-538-113). Further information regarding registration and approval is listed in Supplementary Notes 1 and 2. The study was preregistered (23.01.2015) prior to the inclusion of the first patient (08.07.2015) at the WHO International Clinical Trials Registry Platform. Changes in protocol are detailed in Supplementary Note 2. First patient first visit (FPFV) was on 08.07.2015, last patient in (LPI) was at 25.05.2020 and last patient last visit (LPLV) was at 11.08.2020. The final study report with all planned analyses was submitted on 27.08.2021 to the federal authorities. All participants and if needed their legal representatives provided written informed consent to participate in this trial and all subject-specific trial procedures started after giving consent. This consent included the possibility to share de-identified individual data.

### Randomization and blinding procedure

Participants were recruited at each site by trained members of the study teams. Randomization was block-wise stratified for the treatment groups and trial sites centrally by the Muenchner Studienzentrum (MSZ, Munich Germany) using the RANCODE software^18^. Only MSZ employees who were not otherwise involved in the trial had access to the random allocation sequence. After checking and meeting eligibility criteria, a recruitment form was sent via fax message to the MSZ, and the individual study procedures started. Blinding of all team members and participants was ensured by manufacturing identical capsules and providing identical titration schemes for all treatment groups (Supplementary Notes 3 to 5).

### Study procedures and interventions

**Fig. 1:**
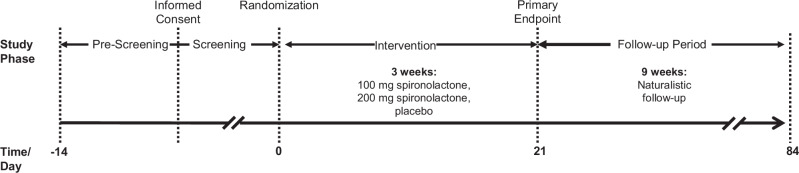
Sequence of trial milestones per patient. This chart shows the trial milestones. After giving written informed consent patients were randomized and treated for three weeks as detailed in the methods section. Primary endpoint was reached three weeks after the initiation of treatment. After reaching the primary endpoint visit, patients entered a nine-week naturalistic follow-up period without study medication.

Supplementary Table 1 provides frequency of study visits and a summary of all study procedures. The intervention period was three weeks followed by a nine-week follow-up period without any study medication (Fig. 1)^18^. After inclusion and baseline visit, participants received study drugs (spironolactone 100 mg or 200 mg) or placebo in addition to the ongoing treatment. The maximal dosage of spironolactone was chosen to be lower compared to the preclinical trial that used 400 mg^19^ with the idea to reduce the titration period and the risk for hyperkalemia especially regarding the combination with potentially QTc-increasing antipsychotics^18^. All concurrent medication was documented in the case report form (CRF). In cases where two antipsychotics were used, a maximum of 1000 chlorpromazine equivalents should not be exceeded and the target symptom for this combination treatment had to be defined precisely. Lorazepam (max. 3 mg/d), lormetazepam (max. 3 mg/d), diazepam (max. 20 mg/d), zopiclone (max. 7.5 mg/d) and biperiden (max. 4 mg/d) were allowed as concurrent medication (e.g., rescue medication)^18^. According to the visit plan participants received during the three-weeks intervention period three times/week blood drawn to test for hyperkalemia. A total of 13 visits were performed for all participants who completed the trial. All details regarding safety monitoring, documentation and data collection as well as the roles of the ethics committees and health authorities have been described previously^18^. All protocol versions are available in German language as well as the statistical analysis plan and can be provided upon request from the first-author and the summary of the trial protocol has been published^18^.

### Study outcomes

The primary endpoint was predefined as change in working memory performance assessed by the n-back test (2-back level) before versus after the intervention period (V10 vs. V1) as detailed elsewhere^18^. A computerized (Presentation Version 16.5, https://www.neurobs.com/) n-back paradigm with three loads (0-back, 1-back, 2-back) was used applying the specifications described elsewhere^18^. We recorded for each load the sums of correct hits, errors, missing responses and reaction times. The correct hit rate was used to calculate the relative hit rate (hits/targets) for each load that was used for further analysis.

Several secondary endpoints were assessed as detailed also elsewhere^18^. These included changes after the intervention and follow-up period in the remaining n-back test results (relative hit rates, error rates, and reaction times). Verbal declarative memory was assessed by the German version of the Rey Auditory Verbal Learning Test (Verbaler Lern- und Merkfaehigkeitstest, VLMT)^32^. Complex visual scanning, motor speed and the ability to shift strategies was assessed by the Trail-Making-Test A (TMT-A and TMT-B)^33^ and the d2-attention test has been used for assessing sustained and selective attention^34^. These cognitive tests were applied to evaluate other aspects of cognition in addition to working memory assessed by the n-back task. Psychopathology was evaluated using the Positive and Negative Syndrome Scale (PANSS)^35^ (including the Andreasen remission criteria), the Calgary Depression Scale for Schizophrenia (CDSS)^36^, the Clinical Global Impression scale (CGI)^37^ and the Global Assessment of Functioning scale (GAF)^38^.

In addition to the assessment of adverse events (AE, see CTCAE Version 4.0), severe adverse events (SAE) and suspected unexpected serious adverse reactions (SUSAR) defined by established definitions and legal requirements, were captured together with several other safety measures^18^. These measures included physical examinations, electrocardiography (ECG), study laboratory (focus on potassium and creatinine levels), blood pressure and heart frequency, assessment of height, body weight and body mass index (BMI)^18^. Motor-side effects were evaluated using the Simpson-Angus scale (SiAS)^39^. We prespecified that during the follow-up period (after V11), hospitalization to a psychiatric hospital due to a worsening of schizophrenia symptoms was not a SAE.

### Statistics and reproducibility

Since no previous studies with this approach were available at the initiation of the trial, we powered the trial using the following assumptions. For primary endpoint analyses using a repeated measures ANOVA (RM-ANOVA) approach, a planned type I error probability of α = 0.05, a desired power of 1 − β = 0.8, three groups (spironolactone 100 mg, 200 mg, placebo), two measurement time points (visit V10 vs. V1), and a between-measurement correlation of *r* = 0.4, medium effects of f = 0.30 or higher can be detected for the within-subject factor time, for the between-subject factor group, and for time x group interactions with a total sample size of 81 (27 participants per group) at V10 (calculations were performed using G*power 3.1.7^40^). As outlined elsewhere^18^, we used multiple analyses on Monte Carlo simulated data for 81 subjects (27 subjects for each of the three groups) at two measurement time points (V1, V10). This was performed using a linear mixed model to confirm sufficient power for this primarily used method. To account for possible dropouts ( ~ 10% during the 3-weeks intervention), we planned to include 90 patients.

Unless otherwise indicated, all statistical analyses presented in this manuscript were pre-specified in the Statistical Analysis Plan (SAP) prior to unblinding. The SAP is available upon request (in German) from the first author. The trial statistician (TSA) remained blinded until data-base hard-lock. The intention-to-treat (ITT) population was defined as all randomized patients who received at least one dose of the investigational medical product (IMP) and was the decisive population for all respective analyses. The per-protocol population (PP) included all subjects evaluable for the primary endpoint without major protocol deviations and the safety population was equal to the ITT-population. IBM SPSS 28 for windows was used for all statistical analyses.

Normality assumption was checked with a Kolmogorov–Smirnov-Test and if this assumption was violated, a Rankit-transformation^41,42^ was performed. For the intention-to-treat population, primary and secondary outcomes were analyzed with a linear mixed model (LMM) as primary analysis method, non-restrictively assuming an unstructured covariance matrix. Group (spironolactone 100 mg, spironolactone 200 mg, placebo) was defined as fixed between-subject-factor and time (1) V1 vs. V10; (2) V1 vs. V10 vs. V12) as within-subject factor. Further fixed factors were gender and site. Covariates were age and duration of education, and subject was included as a random factor. Time effects across visits (V1, V10, V12) were analyzed separately for each of the three groups using linear mixed models. To estimate effect sizes for the time factor and the time × group interaction, repeated-measures ANCOVAs were performed. These used the same covariates as the LMMs—study center, sex, age, and years of education. Secondary endpoints were analyzed in the same manner where appropriate. Here, we focused on the analyses of all timepoints with available data unless otherwise indicated. If there was a significant deviation from normality assumption, a Rankit transformation was again performed. In cases where despite the Rankit transformation the normality assumption was not fulfilled, non-parametric tests were used: Kruskal-Wallis-Tests (3-groups) or Mann-Whitney U-Tests (2-groups) for between group comparisons. Categorical variables were analyzed using Chi²-Tests or adapted tests where needed. Where indicated, a correction for multiple comparisons was performed with the Sidak procedure. AEs and SAEs were classified according to CTCAE V. 4.0, coded according to MedDRA V. 23.1. and evaluated using absolute and relative frequencies. Serious AEs and AEs which are causally related to study medication were tabulated separately. For selective outcomes, two-group comparisons (both intervention groups vs. placebo group) and exploratory sensitivity analyses (excluding the 25% patients with the best 2-back performance prior to treatment at V1 to reduce a potential ceiling effect) were performed. As an additional sensitivity analysis of the primary outcome, we included a set of unplanned generalized linear mixed models (GLMM) using a binomial distribution with a logit link function. The models included group, time and gender as fixed factors, with age, baseline 2-back relative hit rate and years of education entered as covariates. Subject was included as a random factor.

## Results

**Fig. 2:**
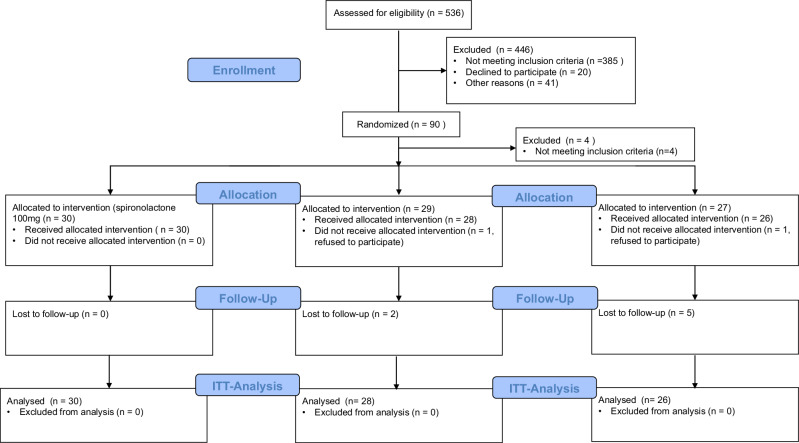
CONSORT flowchart diagram. The diagram shows patient flow through the trial with the number of patients who were randomly assigned, received the intended intervention and were analysed for primary outcome as well as the losses and exclusions after randomization, together with reasons.

We enrolled 90 patients that were randomized to one of the three treatment arms (CONSORT chart, see Fig. 2). 84 patients were investigated as Intention-to-Treat (ITT) population and Safety population. 73 patients were analyzed as Per-Protocol (PP) population (Supplementary Note 3). No differences between groups for the duration of illness, the number of hospitalizations and psychopathological measures could be detected at baseline (see Table 1).

**Table 1:** Demographics and clinical characteristics of the ITT-population at baseline

	Spiro 100 mg				Spiro 200 mg				Placebo				Group comparison		
	number				number				number				LR	df	p
Center (LMU / Regensburg / TUM)	24 / 3 / 3				23 / 1 / 4				22 / 1 / 3				1.48	4	0.830
Gender (male /female)	21 / 9				19 / 9				21 / 5				1.34	2	0.511
Course of the disease (continuous / episodic)	13 / 16				7 / 21				9 / 17				2.49	2	0.288

	Spiro 100 mg				Spiro 200 mg				Placebo				Group comparison		
	*n*	median	min	max	*n*	median	min	max	*n*	median	min	max	F	df	*P*
Age (years)	30	36.5	20	62	28	35.5	19	57	26	35.5	18	58	0.4	2, 81	0.673
Age at first hospitalization (years)	29	25.0	17	56	28	24.0	17	44	26	26.0	16	48	0.68	2, 80	0.507
Duration of Illness (months)	29	72.0	7	432	28	114	8	324	26	114	11	456	0.17	2, 80	0.845
number of hospitalizations	28	3.0	1	30	28	2.0	1	20	25	3.0	1	30	0.49	2, 78	0.612
Duration of School education (years)	29	10.0	8.0	15.0	28	12.0	9.0	17.0	26	11.0	8.0	17.0	1.03	2, 80	0.363
Duration of School Education + Vocational Training (years)	28	13.0	8.0	23.0	27	15.0	9.0	26.0	26	13.0	9.0	23.0	1.95	2, 78	0.149
blood pressure (systolic) baseline	30	121	101	164	28	125	103	160	26	126	96	145	0.43	2, 81	0.649
blood pressure (diastolic) baseline	30	79.5	62	103	28	83.0	62	102	26	82.5	57	96	0.04	2, 81	0.963
Heart rate	30	80.5	61	101	28	83.0	55	120	26	85.5	64	105	0.96	2, 81	0.387
Weight	30	79.1	54.0	114.0	28	87.5	50.3	116	26	88.0	59.0	157.0	2.29	2, 81	0.108
Height	30	172	153	193	28	179	162	191	25	179	158	193	3.35	2, 80	0.040
BMI	30	26.5	17.9	40.4	28	26.4	18.4	38.6	25	28.1	20.9	44.1	0.81	2, 80	0.447
PANSS positive score baseline	30	11.5	7	19	28	12.0	7	20	26	12.0	7	20	0.16	2, 81	0.852
PANSS negative score baseline	30	14.5	7	24	28	13.0	8	25	26	15.0	8	22	0.26	2, 81	0.775
PANSS general score baseline	30	26.5	18	43	28	24.0	16	35	26	27.0	16	38	1.23	2, 81	0.331
PANSS total score baseline	30	50.5	36	72	28	51.5	32	75	26	53.5	33	71	0.56	2, 81	0.576

For categoric variables, groups were compared with likelihood ratio chi-square tests, for continuous variables, groups were compared with one-way analysis of variance (ANOVA). All tests were two-sided without adjustment for multiple comparisons.

*LMU* Ludwig-Maximilians-University, *TUM* Technical University München, *BMI* body mass index, *PANSS* Positive and Negative Syndrome Scale, *n* number of patients, *m* mean, *sd* standard deviation, *LR* likelihood ratio statistic, *F* F statistic, *df* degrees of freedom, *p* *p*-value, *spiro* spironolactone, *mg* milligram.

### Primary outcome measure

**Fig. 3:**
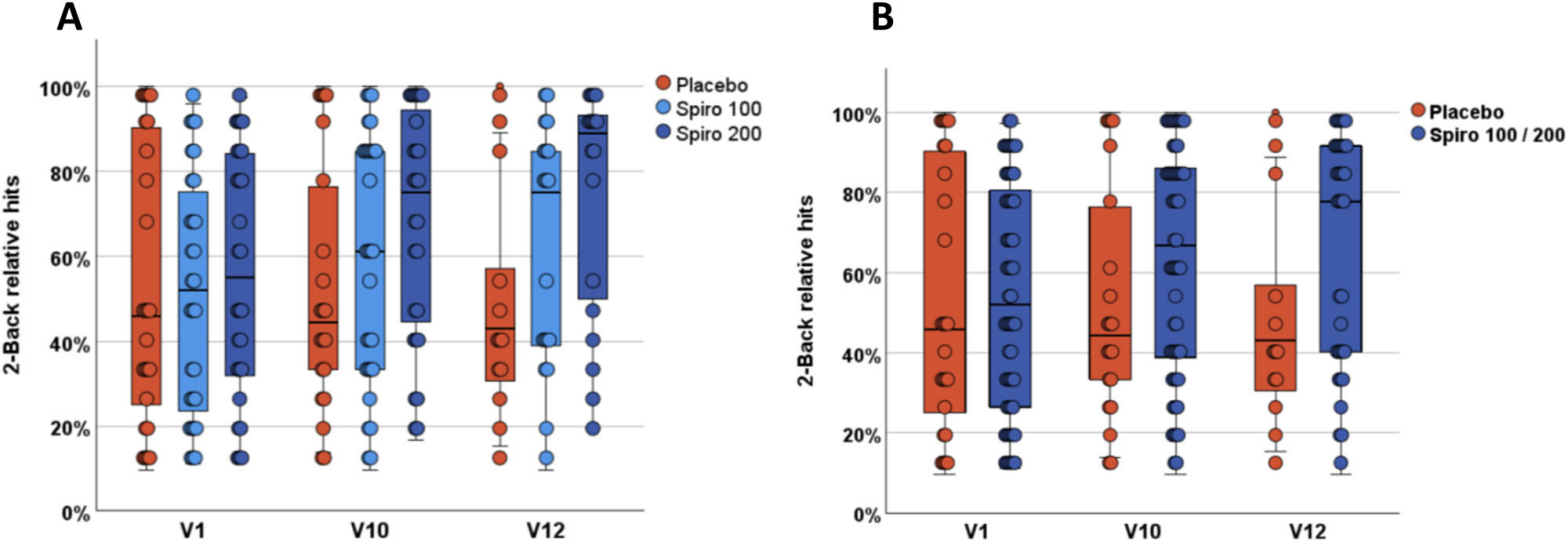
Change in 2-back performance. Boxplots overlaid with individual data points for 2-back performance on all three study groups. Boxes show medians (line in the middle of the boxes), 25%-quartile (lower boundary of the boxes), 75%-quartile (upper boundary of the boxes); Spiro 100: Spironolactone 100 mg, Spiro 200: Spironolactone 200 mg. **A** Placebo group sizes: V = Visit; V1: *n*** =** 26, V10: *n*** =** 21, V12: *n*** =** 13; Spironolactone 100 mg group sizes: V1: *n*** =** 30, V10: *n*** =** 30, V12: *n*** =** 19; Spironolactone 200 mg group sizes: V1: *n*** =** 28, V10: *n*** =** 27, V12: *n*** =** 18; Two-sided Linear Mixed Model (LMM) (factors time, group; interaction time x group, adjusted for site, gender, age and school duration) was performed for Rankit transformed data. This test resulted in significant effects for factor time (*p*** =** 0.00036), but not for interaction between time and group (*p*** =** 0.593). **B** Placebo group sizes: V = Visit; V1: n = 26, V10: *n* = 21, V12: *n* = 13; Spironolactone (combined 100 mg / 200 mg): V1: *n* = 58, V10: *n* = 57, V12: *n* = 37; Two-sided Linear Mixed Model (LMM) (factors time, group; interaction time x group, adjusted for site, gender, age and school duration) was performed for Rankit transformed data. This test resulted in significant effects for factor time (*p *= 0.004), but not for interaction between time and group (*p* = 0.423). Analyses were not adjusted for multiple comparisons.

The primary endpoint analyses in the ITT-population could not establish a significant *time x group* interaction for the comparison of the three study groups between V1 and V10 (F_(2, 74.882)_ = 0.606, *p* = 0.548, effect size f = 0.16) for mean 2-back relative hits. For the comparison of V1, V10 and V12 the *time x group interaction* was still not significant (F_(4, 59.906)=_0.703, *p* = 0.593, effect size f = 0.17). The descriptive data as shown in Fig. 3 indicate a numerical improvement in both intervention groups (100/200 mg spironolactone) – there was a general effect of factor time from the analysis comparing V1 and V10 (F_(1, 75.061)_ = 14.092, *p* < 0.001, effect size f = 0.43) as well as from the analysis comparing V1, V10 and V12 (F_(2, 59.99)_ = 9.079, *p* < 0.001). Outcomes for the primary and secondary endpoint analyses are shown in Supplementary Table 2 (ITT population) and in Supplementary Table 3 (PP population). Supplementary Tables 4 to 7 summarize the post hoc comparisons within groups between visits (V1, V10, V12), where time effects were observed in several outcomes. In particular, the primary outcome – 2-back performance — showed significant improvement over time in the spironolactone 100 mg group (*p* = 0.017, V1 vs. V10: *p* = 0.026, V1 vs. V12: *p* = 0.022, V10 vs. V12: n.s., f = 0.153) and in the spironolactone 200 mg group (*p* = 0.004, V1 vs. V10: *p* = 0.011, V1 vs. V12: *p* < 0.001, V10 vs. V12: n.s., f = 0.297), while for the placebo group no significant time effect was observed.

### Secondary endpoints

Descriptive data and test statistics of the cognitive secondary endpoints and all other secondary endpoints are listed in the supplement (Supplementary Tables 2 and 3 and Supplementary Notes 9– 12). In agreement with the primary endpoint analyses, the LMM of 1-back and 0-back did also not show significant *time x group interactions* (F_(2, 76.810)_ = 0.539, *p* = 0.586; F_(2, 75.473)_ = 0.034 *p* = 0.967). While for 1-back, a significant effect of time (*p* = 0.002; improved for V10 vs. V1) could be observed, this was not detected for 0-back (*p* = 0.173). Extending the 2-back analyses to all three visits (V1, V10 and V12) in the ITT-population there was again no significant *time x group interaction* (*p* = 0.593). There was a general effect of *factor time* (F_(2, 59.993)_ = 9.079, *p* < 0.001). In agreement with the 2-back analyses, also the 1-back (*p* = 0.607) and 0-back (*p* = 0.643) condition showed no significant *time x group interaction* using the Rankit-transformed data (Supplementary Tables 2 – 3).

### Sensitivity analyses (patients receiving spironolactone vs. placebo group)

As a further secondary analysis, we performed a planned LMM for the primary endpoint analysis (relative hits from 2-Back test) comparing all patients who were randomized to spironolactone (100 mg and 200 mg as one group) versus placebo. Again, LMM did not show a significant *time x group interaction* for the comparisons V1 vs. V10 (F_(1, 76.709)_ = 0.602, *p* = 0.440) and V1 vs. V10 vs. V12 (F_(2, 61.859)_ = 0.872, *p* = 0.423). Both analyses showed a group unspecific effect of time (*p* = 0.002; *p* = 0.004) indicating intervention-independent learning effects of the working memory task (Fig. 3).

### Sensitivity analyses (exclusion of the 25% patients with the best n-back performance at baseline)

**Fig. 4:**
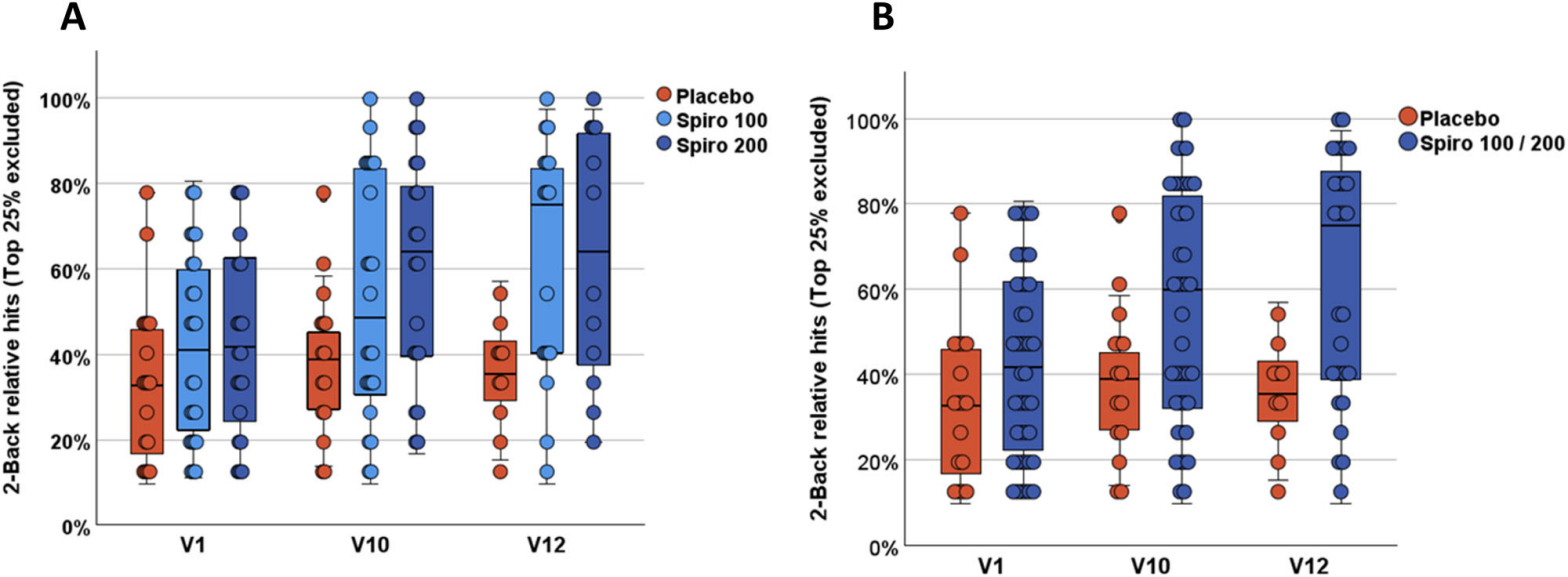
Sensitivity analysis without the 25% of best performing patients. Boxplots overlaid with individual data points for 2-back performance on all three study groups. Boxes show medians (line in the middle of the boxes), 25%-quartile (lower boundary of the boxes), 75%-quartile (upper boundary of the boxes); Spiro 100: Spironolactone 100 mg, Spiro 200: Spironolactone 200 mg, **A** Group sizes: Placebo: V = Visit; V1: *n*** =** 18, V10: *n*** =** 15, V12: *n*** =** 10; Spironolactone 100 mg group sizes: V1: *n*** =** 24, V10: *n*** =** 24, V12: *n*** =** 17; Spironolactone 200 mg group sizes: V1: *n*** =** 20, V10: *n*** =** 19, V12: *n*** =** 12; Two-sided Linear Mixed Model (LMM) (factors time, group; interaction; time x group, adjusted for site, gender, age and school duration) was performed for Rankit transformed data. This test resulted in significant effects for factor time (*p*** =** 0.00002), but not for interaction between time and group (*p*** =** 0.552). **B** Group sizes: Placebo: V = Visit**;** V1: *n* = 18, V10: *n* = 15, V12: *n* = 10; Spironolactone (combined 100 mg / 200 mg): V1: *n* = 44, V10: *n* = 43, V12: *n* = 29; Two-sided Linear Mixed Model (LMM) (factors time, group; interaction; time x group, adjusted for site, gender, age and school duration) was performed for Rankit transformed data. This test resulted in significant effects for factor time (*p* = 0.00044), but not for interaction between time and group (*p* = 0.455). Analyses were not adjusted for multiple comparisons.

We have determined the quartiles for the hit rate in the 2-back at V1. 73.8% of patients had a score of <83 (i.e., exclusion 26.2%), and 77.4% had a score of <84 (i.e., exclusion 22.6%). Thus, we have set the 25% upper limit to 83. For the 2-Back, we have large standard deviations (in many cases >50% from the mean) increasing the noise in all analyses. Comparing V10 to V1, we were able to show an increase in n-back performance in both treatment groups (100 mg: + 25%, 200 mg: +38%, PLC: +13%) without a significant time x group interaction (F_(2, 54.3)_ = 0.677, *p* = 0.512, effect size f = 0.20). Comparing V12 with V1, we were able to show a large increase in performance in the 200 mg group ( + 49%) and 100 mg ( + 46%) compared to placebo ( + 4%) whereas the available sample size at V12 was low (100 mg: *n* = 17, 200 mg: *n* = 12, Placebo: *n* = 10). Pooling both treatment groups, the comparisons V10 to V1 (spironolactone: +31%, PLC: +13%; *p* = 0.59) V12 to V1 (spironolactone: +47%, PLC: +4%; *p* = 0.45) showed a similar pattern (Fig. 4).

### Sensitivity analyses (responder analysis using GLMM)

**Fig. 5:**
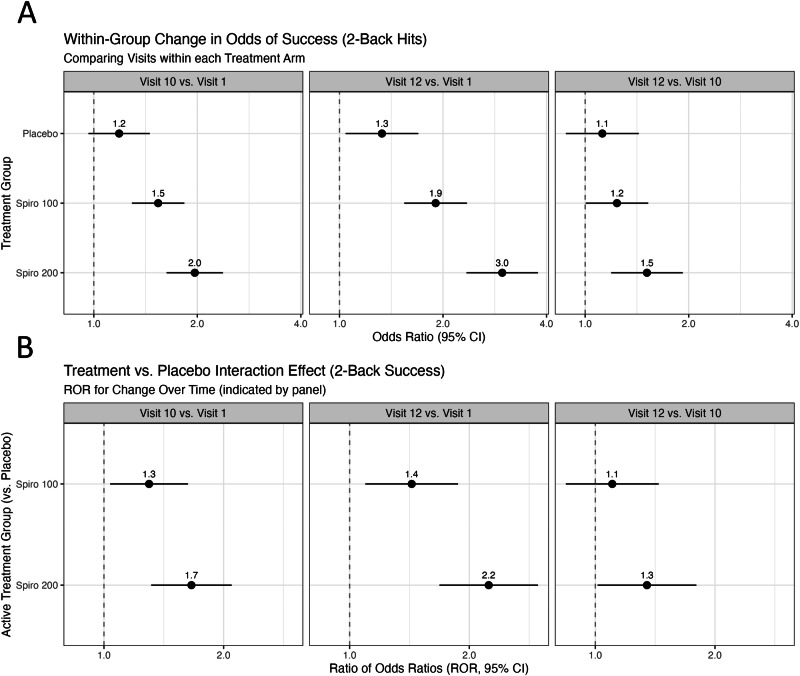
Binomial GLMM analysis of 2-back absolute hits. **A** Odds ratios (OR) and (**B**) ratios of odds ratios (ROR) (vs. placebo) with OR point estimate (**A**) or ROR point estimate (**B**) as center for the error bars and 95% Wald confidence intervals for changes across visits, estimated from a binomial GLMM (logit link) with fixed effects for treatment group, visit, their interaction, baseline 2-back relative hit rate, age, sex, and years of education, and a random intercept for subject. Dashed line: OR/ROR = 1 (no change). All tests two-sided; no multiple comparisons adjustment. ITT population: *n* = 26 (placebo), *n* = 30 (spironolactone 100 mg), *n* = 28 (spironolactone 200 mg), where *n* = number of participants.

To complement the primary analysis, we conducted an unplanned exploratory analysis using a binomial generalized linear mixed-effects model (GLMM), which directly models the number of correct hits out of total trials. The model included fixed effects for treatment group, visit, their interaction, baseline 2-back performance, age, sex, and years of education, and a random intercept for subject. Within-group comparisons showed significant improvements from baseline (V1) to post-intervention (V10) in both spironolactone groups (100 mg: OR = 1.5, *p* < 0.0001; 200 mg: OR = 2.0, *p* < 0.0001), sustained at follow-up (V12: ORs of 1.9 and 3.0, both *p* < 0.0001). The placebo group showed smaller improvement (V12: OR = 1.3, *p* = 0.017). Treatment-by-visit interaction analyses demonstrated that both spironolactone groups showed significantly greater improvement compared to placebo. From baseline to V10, ratio of odds ratios (RORs) were 1.7 (*p* < 0.0001) for 200 mg and 1.3 (*p* = 0.023) for 100 mg. From baseline to V12, RORs were 2.2 (*p* < 0.0001) and 1.4 (*p* = 0.009), respectively. Continued improvement from V10 to V12 was significant for the 200 mg group (ROR = 1.3, *p* = 0.041) but not the 100 mg group (ROR = 1.1, *p* = 0.475) (Fig. 5).

### Safety

The safety profile was in accordance with the previously reported safety profile of spironolactone. Adverse Events and SAEs were classified according to CTCAE V. 4.0 and coded according to MedDRA V. 23.1. A total of 154 AEs and two SAEs (both defined as hospitalization due to worsening of psychosis, moderate, not related, recovery as outcome) were reported in 84 patients. In the ITT sample 152 AEs appeared. Please see Supplement (Supplementary Notes 10–13 and Supplementary Table 8) for all safety outcomes.

## Discussion

SPIRO-TREAT is to the best of our knowledge the first clinical trial aiming to repurpose a pharmacological compound following the rational that the mineralocorticoid receptor antagonist spironolactone would act on the Nrg1-ERBB4 receptor pathway to improve working memory and flexibility learning deficits in patients with schizophrenia^19,31^. Thus, this trial is the first targeting a pathway considered to be part of the druggable genome in schizophrenia. By finalizing the trial, we were able to show that a direct translational approach from preclinical models via animal experiments to clinical research can be realized in one academic setting as suggested by the NIMH^16,17^. Moreover, we were able to show that the application of high dose spironolactone for 3 weeks was safe and well tolerated paving the path for potential follow-up trials exploring e.g., longer treatment periods or higher doses. However, our planned analyses were negative regarding the primary and all secondary endpoints. Against our initial assumptions, the add-on treatment with spironolactone did not result in a significantly improved cognitive performance according to the relative 2-back hit rate compared to a treatment with placebo. However, the descriptive data showed a large numerical effect of spironolactone up to approximately 50% improvement (especially 200 mg or both spironolactone dosages pooled) with only a limited placebo response. Thus, we decided to add an unplanned exploratory analysis using a binomial mixed-effects model specially to account for the relevant individual variability observed in this trial. Here, we were able to show a significant effect of the intervention, especially of spironolactone 200 mg, over placebo.

Targeting CIAS is still challenging with no compound showing sustainable effects in trials or in clinical practice. Recently, Boehringer-Ingelheim’s compound Iclepertin^14^ (GlyT1 inhibitor) failed in a large-scale clinical trial program to show any benefit on cognitive outcome in schizophrenia suffering the same fate as the related compound Bitopertin that was evaluated by Roche some years ago^13^. These prominent failures are just two examples in a long list of failed attempts (other examples include Luvadaxistat) to pharmacologically modulate CIAS. On the other hand, the successful clinical trial with the muscarinic receptor agonist KarXT (xanomeline–trospium) in schizophrenia has opened up new hope towards improving negative and cognitive symptoms in schizophrenia via this novel target mechanism^43^. Unfortunately, this compound was recently not successful in a phase-III add-on trial^44^. However, SPIRO-TREAT differs from those trials as our approach was rooted in a pathway linked to the druggable genome of schizophrenia. When considered alongside the significant numerical improvement observed in our exploratory analysis, these results may pave the way for a future biomarker-stratified trial. In our trial, we aimed at addressing a pathway that has been extensively evaluated in preclinical and animal models^19–28,31^, and we used a strict methodology of assessing cognitive functioning^18^. Moreover, our safety outcomes may allow a follow-up trial to employ higher dosages of spironolactone – in the animal studies a dosage equally 400 mg spironolactone in humans was used^19^. We decided to use only 200 mg for safety reasons, but the outcomes of our trial and the finding that our 200 mg dosage was more effective than 100 mg would justify higher dosages in a potential follow-up trial. In that regard, one, internationally non-registered underpowered trial provided spironolactone 100 mg for eight weeks in schizophrenia patients in addition to risperidone and showed a beneficial effect on psychopathology according to PANSS without reporting cognitive outcomes^45^.

Many patients in the presented study received guideline-based antipsychotic treatment. Hence, we cannot rule out that specific drug interactions may have modulated the impact of spironolactone as adjuvant treatment. Of note, in a highly controlled pre-clinical setting spironolactone improved flexibility learning in a 2-hit mouse model (*Tcf4* transgenic mice subjected to psychosocial stress). Nonetheless, an add-on treatment with aripiprazole negatively interfered with spironolactone’s impact on cognition^46^. If this poses a general negative drug interaction mediated by all or specific subgroups of FGA and/or SGA antipsychotics currently remains unclear and requires further targeted research. However, phospho-Erbb4 levels were not determined in *Tcf4* transgenic mice, leaving it unclear whether the (NRG1)-ERBB4 pathway is modulated in this mouse model of schizophrenia^18,19^. When targeting ERBB4 in the brain, it is critical that compounds are selective and do not antagonize other members of the ERBB receptor family, in particular the epidermal growth factor receptor (EGFR), as EGFR is crucial for the propagation of neural precursor cells and their differentiation into neurons^47^. Spironolactone displayed a roughly fivefold selectivity for ERBB4 over EGFR and showed a mild antagonism on the serotonin receptor 2 A (HTR2A)^19,46^, possibly explaining the mild, but not significant effects we observed in this study. Therefore, the development of highly selective ERBB4 kinase inhibitors with an appropriate polypharmacological profile on other schizophrenia-relevant receptors (e.g., antagonistic on receptors DRD2, HTR2A, but agonist on M1 and M4 muscarinic receptors) might be a promising route for an effective treatment. In this vein, members of our group have identified a selective ERBB4 antagonist with an eightfold selectivity over EGFR and mild antagonism against HTR2A in a cell-based profiling assay^46^.

We have already described the important limitations of our trial. However, as this was the first attempt with this concept, it was designed not only to test efficacy but also to acknowledge safety. From a pharmacological perspective one may assume that spironolactone was not specific enough to interact perfectly with the target pathway. Here, compounds that act more specifically on (NRG1)-ERBB4 pathway, such as novel ERBB4 selective kinase inhibitors as described elsewhere^46^, may be more effective. Finally, due to the covid pandemic and change of institutions of the key investigators, we had a delay in submitting the results. However, the final report with all planned analyses was published on time to the regulatory authorities.

## Conclusions

In summary, we present the first trial of spironolactone to antagonize (NRG1)-ERBB4 signaling pathway for the treatment of cognitive symptoms in schizophrenia. Our findings indicate that spironolactone may be a potential add-on treatment for CIAS. However, more research to confirm our exploratory findings is needed prior to an application in clinical practice. The favorable safety profile and the reported improvements, particularly in the spironolactone 200 mg group, support further evaluation of this approach, including higher spironolactone dosages or longer trial durations.

## Supplementary information

Transparent Peer Review file

Supplementary Material

Description of Additional Supplementary Files

Supplementary Data 1

Supplementary Data 2

## Data Availability

All source data (means and individual data) underlying the main analyses, graphs and charts are available as Supplementary Data. The supplementary Data Set 1 includes anonymized individual patient data to understand the main outcome analyses presented in this publication. The supplementary Data Set 2 contains all values for the development of the figures here presented. The Supplemental Material contains all information regarding ethics approvals and approvals of the legal authorities, compliance, protocol violations, safety assessments, the frequency and scope of the study visits, a detailed overview of the study treatment (including batch numbers), supplementary results with secondary outcomes, further result tables as indicated in the manuscript and AE as well as SAE listings. Further relevant data, including all SAE details, is available from the authors as anonymous data upon request. Please sent your request to the first author alkomiet.hasan@med.uni-augsburg.de and to the CRO (muenchner.studienzentrum@mri.tum.de). All requests will be verified within four weeks after first request and data will be provided after approval of the trial statistician (TSA) and responsible PIs (A.H., P.F.).
