# Supplementary material for: Antagonizing NRG1-ERBB4 signaling pathway with spironolactone for the treatment of schizophrenia: results of a randomized controlled drug repositioning clinical trial
Source: Commun Med (Lond). 2026 Jun 16;6:337. doi: 10.1038/s43856-026-01714-3 (PMC13272664; doi:10.1038/s43856-026-01714-3)
Supplement: Supplementary file 2 — Supplementary Material [file 43856_2026_1714_MOESM2_ESM.pdf]

Supplementary Material (in parts derived from the published final study report)

Supplementary Note 1

Study period

First patient first visit (FPFV): 08.07.2015; Last patient in (LPI): 25.05.2020; Last patient last visit (LPLV): 11.08.2020

Supplementary Note 2

Approvals and Amendments

Approval: Bundesinstitut für Arzneimittel und Medizinprodukte (BfArM): 23.02.2015; Ethics Committee (EC): 11.03.2015.

Clinical Study Protocol (CSP) Version 1.0, 07.01.2015; Informed Consent Form (ICF) Version 07.01.2015

Amendments (AM) and changes in conduct to the clinical trial (AM1 - AM7)

Approval AM1: BfArM: 08.04.2015; EC: 02.04.2015

The following major changes were included in AM 1: Prof. Falkai is SDP site 1, SOFAS assessments have been removed from study visits as it is the same examination as GAF assessments; description of inclusion & exclusion criteria as in CSP under 4.2 and 4.3. The first patient was included after approval of AM 1. CSP Version 2.0, 12.03.2015; ICF Version 1.0 07.01.2015

Approval AM 2: BfArM: 30.09.2015; EC: 15.10.2015

The following major changes were included in AM 2: extension of inclusion criterion 3 (not only patients with monotherapy, but with two antipsychotics allowed) and clearer definition of inclusion criterion 5 and exclusion criterion 8. CSP Version 3.0, 22.09.2015; ICF Version 1.1, 21.09.2015

Approval AM 3: BfArM: 11.10.2016; EC: 04.10.2016

The following major changes were included in AM 3: additional inclusion of women allowed (not pregnant, contraception according to CTFG guideline), clearer definition of excluded antipsychotics. CSP Version 4.1, 19.09.2016; ICF Version 2.0, 06.09.2016

Approval AM 4: BfArM: 18.04.2017; EC:18.04.2017

The following major changes were included in AM 4: Additional site Regensburg, clarification of the negative wording of an exclusion criterion, extension of study period

CSP Version 4.2, 23.02.2017

AM 5: EC: 24.04.2018 (submission to BfArM for information: 21.03.2018)

The following non-substantial changes were included in AM 5: clarification that exclusion of anamnestic epileptic seizures concerns only subjects participating in TMS part of the clinical trial.

CSP Version 4.3. 08.02.2018

Approval AM 6: BfArM: 25.06.2018; EC: 25.06.2018

The following major changes were included in AM 6: change in IMP Manufacturer and add-on to patient information to reflect new regulation on data protection

CSP Version 4.4, 23.05.2018

Approval AM 7: BfArM: 31.10.2019; EC: 08.11.2019

The following major changes were included in AM 7: adapted description in power planning and statistical analysis and improved specification of secondary endpoints
CSP Version 4.5, 17.10.2019

Supplementary Note 3

Compliance

There were five violations of inclusion or exclusion criteria. In subject 121, one inclusion criterion was violated (substance dependency with substance free interval of only four instead of six months). In subject 124, antipsychotic medication was stable for less than seven days. In subject 138, a syncope was retrospectively detected in the patient chart. In subject 157, the cumulative CPZ dose was exceeded. In patient 303, the signature of a MD not involved in the study, was made after the begin of the intervention. All these violations were rated as major protocol violations without being a risk for patient safety or data integrity.

In total, **90 patients** were included (signed informed consent). **4 patients** were not randomized, and **2 patients** were randomized, but did not receive an IMP. Thus, a total of **84 patients** were included in the ITT set. **7 patients did not reach V10**. **11 patients** were excluded from the ITT Population due to major protocol deviations resulting in a total of **73 patients** for the PP population.

Regarding EOS (V12): 5 patients discontinued the study due to an AE, 6 patients discontinued the study due to protocol violations, 18 patients discontinued the study due to lost-to-follow-up, four patient withdrew consent and three patients had other reasons for discontinuing the study. 0 patients stopped treatment due to death or due to a worsening of symptoms.

Supplementary Note 4

Protocol Violation (PV)

154 PV were reported in 69 out of 90 patients. 14 protocol violations in 11 patients were rated as major and the remaining protocol deviations were rated as minor. 86 out of 140 minor protocol violations were due to visits scheduled at other visit timepoints or not-performed visits. 23 out of 140 PV were due to uncomplete assessment of questionnaires or other items (e.g. laboratory data, ecg). 31 minor PV were due to other reasons.

Supplementary Note 5

Study medication

6 patients left the study before intake of any IMP. Further 7 patients received IMP but did not reach visit 10 (V10). From these 7 patients, 5 were randomized to placebo and two to spironolactone 200 mg. Drug accountability was performed as part of all study visits as indicated in table 1 of the main manuscript.

Supplementary Note 6

Safety Assessments (all patients included)

Annual Safety Reports have been provided to PEI and EC for the following periods:
DSUR 1: 23.02.2015 – 22.02.2016

DSUR 2: 23.02.2016 – 22.02.2017
DSUR 3: 23.02.2017 – 22.02.2018
DSUR 4: 23.02.2018 – 22.02.2019
DSUR 5: 23.02.2019 – 22.02.2019
DSUR 6: 23.02.2020 – 11.08.2020

Supplementary Table 1: Frequency and scope of study visits

Study Visit		V1	V2	V3	V4	V5	V6	V7	V8	V9	V10	V11	V12
**Phase**	**Screening**	**Baseline**	**Intervention**									**Post Intervention**	**Close-Out**
**Day**	**-14 bis -1**	**0**	**2**	**5**	**7**	**9**	**11**	**14**	**16**	**19**	**21**	**25**	**84**
Inclusion/Exclusion criteria	X	X											
MINI-Plus interview	X												
Informed consent	X												
Demography		X											
Psychiatric history		X											
Medical history		X											
Randomization		X											
(Serious) adverse events		X	X	X	X	X	X	X	X	X	X	X[1]	X
n-Back		X									X		X
Neuropsychology (VLMT, TMT, d2)		X									X		X
SiAS		X									X		X
PANSS	X	X	X		X			X			X		X
CDSS		X									X		X
CGI		X	X		X			X			X		X
GAF		X									X		X
ECG		X									X		X
Physical examination		X	X								X		X
Vital signs (BP, HR)	X	X	X		X			X			X	X	X
BMI		X									X		X
Study laboratory		X	X	X	X	X	X	X	X	X	X		X
Pregnancy test		X											
Dispense study medication		X			X			X					
Return study medication					X			X			X		

MINI-Plus: MINI-Plus Interview for ICD-10 and DSM-IV diagnosis; PANSS: Positive and Negative Syndrome Scale in Schizophrenia; CDSS: Calgary Depression Rating Scale for Schizophrenia; CGI: Clinical Global Impression; GAF: Global Assessment Scale of Functioning; SiAS: Simpson Angus Scale for EPMS, ECG: electrocardiogram; BP: blood pressure; HR: heart rate; BMI: Body Mass Index, VLMT: Verbaler Lern- und Merkfähigkeitstest; TMT: Trail-Making-Test; d2: d2-attention test, TMS: transcranial magnetic stimulation; (S)AE: (Serious) Adverse Event; [1]Hospitalization to a psychiatric hospital is not defined as SAE after V11. Study laboratory at baseline and V11: sodium, potassium, calcium, creatinine, glomerular filtration rate (GFR), c-reactive protein, aspartate transaminase (AST), alanine transaminase (ALT), gamma glutamyltransferase (GGT), blood count, prothrombin time, partial thromboplastin time; study laboratory V2 to V10: sodium, potassium, creatinine, blood count; study laboratory V12: sodium, potassium, calcium, creatinine

Supplementary Note 7
Information for study treatment procedures

Spironolactone, Spironolacton Hexal encapsulation (1x50 or 2x50mg per capsule)

Dose intended per day**:** In group I (spironolactone 100 mg), at day (D) 1 capsule with 50 mg spironolactone and 1 capsule with placebo, from D2 to D21 two capsules with 50 mg spironolactone (100 mg in total) per day. In group II (spironolactone 200 mg), at D1 one capsule with 50 mg spironolactone and one capsule with placebo, at D2 two capsules with 50 mg spironolactone (100 mg in total), at D3 one capsule with 50 mg spironolactone and one capsule with 100 mg spironolactone (150 mg in total) and from D4 to D21 two capsules with 100 mg spironolactone per day (200 mg in total). In group III (placebo), two capsules with placebo from D 1 to D 21.

Route: oral use

Placebo capsules Bulk: 20150508A, 20160414A, 20170320A, 20170802P, 20180419P, 20180719P, 20190326B, 20200220P

Spironolactone 50 mg Capsules Bulk:

Internally Batch:	Batch finished IP:
20150508B,	Osyrol 50mg 124071/A3
20160415B,	Osyrol 50 mg 124071/A3
20170327A,	Osyrol 50 mg 124071/A3
Internally Batch:	Batch finished IP:
20170803C,	Osyrol 50 mg 154501/A2
20180419C,	Osyrol 50 mg 154501/A2
20180716A,	Spironolacton Hexal 50 mg HX4993
20190326A,	Spironolacton Hexal 50 mg JP9670
20200220A,	Spironolacton Hexal 50 mg JY3667

Spironolactone 100 mg Capsules:

Internally Batch:	Batch finished IP:
20150508C,	Osyrol 50mg 124071/A3
20160415C,	Osyrol 50 mg 124071/A3
20170328B,	Osyrol 50 mg 124071/A3
20170807A,	Osyrol 50 mg 154501/A2
20180419D,	Osyrol 50 mg 154501/A2
20180720B,	Spironolacton Hexal 50 mg HX4993
20190322A,	Spironolacton Hexal 50 mg JP9670
20200220B,	Spironolacton Hexal 50 mg JY3667

Supplementary Note 8

Supplementary results

Secondary Outcomes

Apart from the variables VLMT WF, CDSS and CGI all secondary clinical outcome variables were normally distributed or fulfilled this assumption after Rankit transformation. All LMM analyses showed, apart from a significant *time x group interaction* for GAF, no significant effect. GAF analyses showed also a significant effect of the *factor time* (all $p<0.001$). The interaction was mainly driven by a subtle improvement in the spironolactone 100 mg group (V1 vs. V10), but as the mean increase in GAF is below 10 points, this effect is not clinically relevant.

From the analysis of remaining neuropsychologic tests, we found no significant *time x group interaction* for VLMT (all $p>0.368$), for TMT (all $p>0.191$) and for d2 test (all $p>0.174$) for the V1 vs. V10 comparison, nor for the V1 vs. V10 vs. V12 comparison (VLMT: all $p>0.598$, TMT: all $p>0.177$, d2 test: all $p>0.128$) (see Supplementary results table 2). For VLMT recognition, nonparametric Kruskal-Wallis tests revealed no significant group differences at V1, V10 or V12 (all $p>0.058$). For CGI severity score, Kruskal-Wallis tests showed no significant group effects at any visit (all $p>0.119$). For CDSS Kruskal-Wallis tests showed significant group effects only at V1 ($p=0.024$), following Mann-Whitney U-tests resulted in a significantly larger CDSS score in the Spironolactone 100 compared to the Spironolactone 200 group (Sidak-corrected $p=0.036$).

As PANSS was assessed more frequently during the study course than the cognitive secondary endpoints, PANSS outcomes are presented separately. Please see supplement paragraph 3 and supplementary figure 1 for the course of the PANSS values. For all PANSS analyses no significant *time x group interaction* could be observed in the LMM analyses ($PANSS_{Positive}$: $F_{(12,\ 72.495)}=1.100$, $p=0.374$; $PANSS_{Negative}$: $F_{(12,\ 71.851)}=1.015$, $p=0.445$; $PANSS_{General}$: $F_{(12,\ 67.873)}=1.097$, $p=0.377$; $PANSS_{Total}$: $F_{(12,\ 72.261)}=1.518$, $p=0.138$). In general, a subtle decrease in all PANSS values over the trial period was observed. This pattern did not show any differences across groups and can be expected in such trials as being in a trial provides social and emotional support for study patients by the research teams resulting in a secondary improvement of psychopathology.

Based on PANSS, the RSWG remission criteria (Andreasen criteria) were calculated. No group differences in the frequency of symptomatic remission according to this criterion were observed at V10 ($Chi^2_{(2)}=1.169$, $p=0.557$) and at V12 ($Chi^2_{(2)}=3.632$, $p=0.163$).

Safety Outcomes

While not reaching significance ($p=0.088$), numeric distribution indicates that in both study groups more AEs occurred with no significant differences across these two groups compared to the placebo group. 121 AE were rated Grade 1 (mild), 27 Grade 2 (moderate), 4 Grade 3 (severe), 0 Grade 4 (life-threatening), 0 Grade 5 (death). No significant differences could be observed across study groups ($p=0.164$). Action taken in relation to AE showed again no between-group differences ($p=0.290$). A total of 2 SAEs (2 terms) were reported in 2 patients and no significant differences could be observed across groups ($p=0.565$). Suspected Serious Adverse Reactions (SARs) and SUSARs were not reported

in the study. As expected, spironolactone resulted in a decrease of sodium and an increase of potassium in the spironolactone groups. These effects were observed during the intervention phase, but as expected not during the follow-up phase. See supplement paragraphs 4 - 7 for further details and a listing of all safety parameters and safety outcomes.

Per Protocol analyses

As predefined in our protocol and the statistical analyses plan, ITT-analyses presented above were the main analyses for this study. However, we also performed analyses on the PP-population (all subjects evaluable for the primary endpoint without major protocol deviations). For the primary endpoint analyses, a repeated-measures analysis of covariance (ANCOVA) with the same covariates used in the LMM was applied. These analyses confirmed the negative finding from the LMM analyses in the ITT-population. Again, no significant time x group interaction was observed for the V1 vs. V10 contrast ($F_{(2, 60)}$=0.141, p=0.869).The effect size of this interaction is *partial* η^2=0.005 (related to f=0.07). Due to the negative outcome of the study with no relevant between group-differences for any of the outcome or safety variables, we limited the detailed presentation of per protocol analyses for this report to the primary endpoint analyses. All other per protocol analyses did not reveal any relevant differences compared to the ITT-population regarding efficacy or safety outcomes.

Item	V1			V10			Time x Group Interaction #		
	100 (n=30)	200 (n=28)	PLC (n=26)	100 (m=30)	200 (n=27)	PLC (n=21)	Df	F	p
2-Back relative hits *	51.39±27.63	56.05±28.23	52.56±32.78	59.35±29.48	67.34±27.77	52.35±29.33	2, 74.882	0.606	0.548
1-Back relative hits	64.74±29.80	70.01±31.34	65.19±34.09	71.15±28.24	79.68±25.08	68.62±28.45	2, 76.810	0.539	0.586
0-Back relative hits	89.09±19.42	96.39±4.99	91.67±15.62	93.73±9.10	95.50±12.28	96.03±4.69	2, 75.473	0.034	0.967
VLMT 5th trial	11.20±2.310	11.64 ± 2.93	10.32±2.81	11.37±2.74	11.30±3.28	11.09±2.76	2, 74.870	1.014	0.368
VLMT 7th trial	8.43±3.42	9.68±4.08	8.08±3.43	8.33±3.89	9.54±4.00	8.45±3.74	2, 75.016	0.377	0.687
VLMT trial 5 minus 7	2.80±2.34	1.79±1.97	2.20±1.71	3.07±264	1.81±1.67	2.68±2.19	2, 76.428	0.086	0.918
VLMT triaL 1 to 5	45.43±9.76	49.86±14.34	42.36±13.02	46.73±10.19	49.67±14.13	46.82±12.40	2, 75.771	0.989	0.412
TMT A [sec]	39.57±19.04	36.07±15.63	34.69±13.59	32.97±12.86	29.56±11.40	31.32±10.50	2, 77.136	0.823	0.443
TMT B [sec]	100.13±67.09	87.82±41.83	107.08±57.16	93.83±48.23	74.48±35.22	96.68±47.77	2, 75.744	0.985	0.378
TMT B-A [sec]	60.57±58.11	51.75±33.58	72.38±46.09	60.87±38.74	44.93±26.58	65.55±41.14	2, 76.591	1.692	0.191
d2 total signs	393.48±111.84	431.25±91.93	395.44±118.14	408.69±115.08	466.30±90.14	429.57±100.26	2, 72.358	1.794	0.174
d2 failures	23.97±20.62	26.11±18.10	28.04±35.13	19.62±14.02	22.07±15.31	20.62±19.45	2, 72.944	0.032	0.969
d2 failure %	6.28±6.42	6.07±4.92	6.45±7.13	5.02±4.20	4.83±3.33	5.13±5.69	2, 72.116	0.032	0.969
d2 total minus failures	366.07±105.00	409.07±84.32	364.48±106.55	388.90±114.64	444.56±89.78	408.95±102.25	2, 72.200	1.201	0.307
d2 concentration score	141.97±47.20	156.25±42.46	139.88±61.24	157.52±48.30	176.67±43.88	152.19±60.73	2, 72.988	0.435	0.649
GAF	56.90±9.04	61.75±10.47	57.96±7.37	63.30±10.93	63.70±11.49	59.45±9.76	2, 75.078	5.619	0.005*
CDSS	3.82±3.30	2.07±3.07	2.81±2.19	3.10±2.87	2.15±2.80	3.68±4.67	See main text		
CGI	3.97±0.67	3.75±0.70	4.00±0.63	3.97±0.67	3.65±0.89	3.88±0.73	See main text		
PANSS positive sumscore	12.03 ± 3.65	11.93 ± 3.05	12.27 ± 3.45	10.87 ± 3.25	11.15 ± 3.97	11.50 ± 3.64	2, 75.367	0.057	0.945
PANSS negative sumscore	15.07 ± 5.86	14.18 ± 4.31	14.88 ± 3.28	12.83 ± 4.33	12.33 ± 3.55	14.68 ± 3.23	2, 79.172	1.018	0.366
PANSS general sumscore	27.62 ± 6.76	25.14 ± 5.10	26.92 ± 5.99	24.57 ± 5.81	24.50 ± 5.57	26.14 ± 6.66	2, 77.039	1.108	0.335
PANSS total score	54.72 ± 13.23	51.25 ± 10.70	54.08 ± 10.87	48.27 ± 11.94	47.78 ± 11.22	52.32 ± 11.11	2, 76.698	0.925	0.401

Supplementary Table 2: Main outcomes for ITT-sample V1 vs. V10; results from Linear Mixed Model (LMM; factors time, group, center, gender; covariates age, education;
*primary endpoint. df: degrees of freedom; F: F statistic; p: p-value; CDSS: Calgary Depression Rating Scale for Schizophrenia; CGI: Clinical Global Impression; GAF: Global Assessment Scale of Functioning; VLMT: Verbaler Lern- und Merkfähigkeitstest; TMT: Trail-Making-Test; d2: d2-attention test (d2 F: failures; d2 F%: failure percent; d2 GZ-F: total score minus errors; d2 KL: concentration; # where necessary, analysis were performed on rankit transformed variables

Item	Difference V1 – V10			ANOVA, factor group #		
	Spiro 100 (n=26)	Spiro 200 (n=25)	PLC (n=18)	df	F	p
2-Back relative hits*	-6.84±12.98	-10.33±23.7	-3.4±16.74	2, 52	0.42	0.660
1-Back relative hits	-5.77±18.12	-8.46±25.47	-6.84±16.79	2, 52	0.46	0.636
0-Back relative hits	-4.62±17.19	1.24±14.1	-5.42±17.59	2, 52	0.19	0.827
VLMT 5th trial	-0.15±1.46	0.16±1.93	-0.44±2.5	2, 52	0.17	0.174
VLMT 7th trial	0.04±2.76	0.25±2.71	-0.11±2.91	2, 51	0.72	0.490
VLMT trial 5 minus 7	-0.19±2.97	-0.08±2.04	-0.44±2.62	2, 51	0.41	0.669
VLMT triaL 1 to 5	-1.54±6.65	0.08±10.25	-1.00±8.04	2, 52	2.83	0.065
TMT A [sec]	7.88±9.93	5.4±10.61	2.63±8.98	2, 52	0.69	0.508
TMT B [sec]	7.27±45.38	7.28±26.98	-2.00±28.93	2, 52	6.097	0.004
TMT B-A [sec]	-0.62±48.75	1.88±26.72	-4.63±27.83	2, 52	2.77	0.072
d2 total signs	-17.25±38.22	-26.36±39.91	-34.89±50.39	2, 51	0.04	0.963
d2 failures	5.5±10.98	5.96±14.06	2.39±19.4	2, 51	0.09	0.910
d2 failure %	1.78±2.9	1.49±4.21	0.19±6.36	2, 51	0.09	0.907
d2 concentration score	-14.67±14.91	-18.64±16.11	-7.94±52.47	2, 51	0.06	0.937
GAF	-6.58±6.99	-1.64±5.81	-2.16±7.67	2, 52	1.57	0.218
CDSS	0.79±2.69	-0.04±2.81	-0.84±3.7	See text		
CGI	0±0.28	0±0.51	0.1±0.3	See text		
PANSS positive	1.12±2.6	0.96±2.78	0.32±2.96	2, 52	0.33	0.720
PANSS negative	2.28±6.5	2.16±3.41	0.37±2.85	2, 52	1.87	0.165
PANSS general	3.28±7.49	1.28±3.84	0.42±5.74	2, 52	0.07	0.936
PANSS total	6.68±14.44	4.4±7.83	1.11±9.7	2, 52	0.48	0.622

Supplementary Table 3: Main outcomes for per-protocol sample for differences between V1 and V10; results from ANCOVA (factors group, center, gender; covariates age, education);

*primary endpoint. df: degrees of freedom; F: F statistic; p: p-value; CDSS: Calgary Depression Rating Scale for Schizophrenia; CGI: Clinical Global Impression; GAF: Global Assessment Scale of Functioning; VLMT: Verbaler Lern- und Merkfähigkeitstest; TMT: Trail-Making-Test; d2: d2-attention test (d2 F: failures; d2 F%: failure percent; d2 GZ-F: total score minus errors; d2 KL: concentration.

where necessary, analysis were performed on rankit transformed variables

			95% CI for mean difference		Factor time			
		Mean difference#	Lower bound	Upper bound	F	df	p	Effect size f
**2-Back relative hits ***								
Spiro 100 mg	- V1 vs. V10 vs. V12				4.93	2, 21.96	0.017	0.153
	V1 vs. V10	-0.28	-0.52	-0.04			0.026	
	V1 vs. V12	-0.38	-0.71	-0.06			0.022	
	V10 vs. V12	-0.11	-0.47	0.26			0.551	
Spiro 200 mg	- V1 vs. V10 vs. V12				6.77	2, 31.22	0.004	0.297
	V1 vs. V10	-0.43	-0.75	-0.11			0.011	
	V1 vs. V12	-0.57	-0.88	-0.25			< 0.001	
	V10 vs. V12	0.14	-0.08	-0.36			0.210	
Placebo	- V1 vs. V10 vs. V12				1.15	2, 16.07	0.340	
**1-Back relative hits ***								
Spiro 100 mg	- V1 vs. V10 vs. V12				2.89	2, 22.35	0.077	
Spiro 200 mg	- V1 vs. V10 vs. V12				3.93	2, 28.82	0.031	0.266
	V1 vs. V10	-0.35	-0.64	-0.06			0.019	
	V1 vs. V12	-0.48	-0.85	-0.12			0.010	
	V10 vs. V12	0.13	-0.12	0.38			0.285	
Placebo	- V1 vs. V10 vs. V12				1.34	2, 15.88	0.291	

Supplementary Table 4: Post hoc time effects separately for groups (N-back data): * based on Rankit transformed variables; # based on estimated marginal means; post hoc analysis between timepoints listed only, if main effects for the respective group was significant

			95% CI for mean difference		Factor time			
		Mean difference#	Lower bound	Upper bound	F	df	p	Effect size f
**TMT A ***								
Spiro 100 mg	- V1 vs. V10 vs. V12				9.87	2, 23.56	< 0.001	0.182
	V1 vs. V10	0.37	0.17	0.56			< 0.001	
	V1 vs. V12	0.38	0.16	0.61			0.002	
	V10 vs. V12	0.02	-0.22	0.26			0.874	
Spiro 200 mg	- V1 vs. V10 vs. V12				10.60	2, 23.09	< 0.001	0.293
	V1 vs. V10	0.45	0.16	0.74			0.004	
	V1 vs. V12	0.58	0.30	0.86			< 0.001	
	V10 vs. V12	0.13	-0.16	0.43			0.371	
Placebo	- V1 vs. V10 vs. V12				1.21	2, 15.73	0.323	
**TMT B ***								
Spiro 100 mg	- V1 vs. V10 vs. V12				0.44	2, 23.08	0.650	
Spiro 200 mg	- V1 vs. V10 vs. V12				17.96	2, 39.87	< 0.001	0.248
	V1 vs. V10	0.27	0.03	0.51			0.030	
	V1 vs. V12	0.52	0.32	0.71			< 0.001	
	V10 vs. V12	0.25	-0.05	0.55			0.101	
Placebo	- V1 vs. V10 vs. V12				0.98	2,20.29	0.392	
**TMT B minus A ***								
Spiro 100 mg	- V1 vs. V10 vs. V12				0.76	2, 25.28	0.478	
Spiro 200 mg	- V1 vs. V10 vs. V12				6.76	2, 21.01	0.005	0.157
	V1 vs. V10	0.08	-0.26	0.42			0.617	
	V1 vs. V12	0.42	0.17	0.68			0.004	
	V10 vs. V12	0.34	-0.05	0.73			0.086	
Placebo	- V1 vs. V10 vs. V12				0.82	2, 17.70	0.456	
**d2 - concentration**								
Spiro 100 mg	- V1 vs. V10 vs. V12				9.60	2, 18.44	0.001	0.163
	V1 vs. V10	-13.39	-20.15	-6.64			< 0.001	
	V1 vs. V12	-14.90	-31.12	1.31			0.069	
	V10 vs. V12	-1.51	-18.28	15.25			0.852	
Spiro 200 mg	- V1 vs. V10 vs. V12				42.31	2, 20.73	< 0.001	0.322
	V1 vs. V10	-18.56	-24.94	-12.18			< 0.001	
	V1 vs. V12	.33.69	-42.40	-24.97			< 0.001	
	V10 vs. V12	-15.13	-24.87	-5.39			0.004	
Placebo	- V1 vs. V10 vs. V12				1.57	2, 17.01	0.237	

Supplementary Table 5: Post hoc time effects separately for groups (further cognitive outcomes): * based on Rankit transformed variables; # based on estimated marginal means; post hoc analysis between timepoints listed only, if main effects for the respective group was significant

			95% CI for mean difference		Factor time			
		Mean difference#	Lower bound	Upper bound	F	df	p	Effect size f
**GAF ***								
Spiro 100 mg	- V1 vs. V10 vs. V12				15.00	2, 22.51	< 0.001	0.333
	V1 vs. V10	-0.65	-0.93	-0.37			< 0.001	
	V1 vs. V12	-0.60	-0.96	-0.24			0.002	
	V10 vs. V12	0.05	-0.37	0.46			0.802	
Spiro 200 mg	- V1 vs. V10 vs. V12				1.89	2, 21.16	0.176	
	V1 vs. V10						0.228	
	V1 vs. V12						0.110	
	V10 vs. V12						0.366	
Placebo	- V1 vs. V10 vs. V12				4.23	2, 17.28	0.032	0.199
	V1 vs. V10	-0.22	-0.50	0.06			0.121	
	V1 vs. V12	-0.61	-1.11	-0.11			0.021	
	V10 vs. V12	-0.39	-0.93	0.15			0.145	

Supplementary Table 6: Post hoc time effects separately for groups (Global assessment of functioning (GAF) data): * based on Rankit transformed variables; # based on estimated marginal means; post hoc analysis between timepoints listed only, if main effects for the respective group was significant

			95% CI for mean difference		Factor time			
**PANSS positive** * &								
Spiro 100 mg	- V0V1V2V4V7V10V12				1.24	6, 30.66	0.313	
Spiro 200 mg	- V0V1V2V4V7V10V12				7.16	6, 26.17	< 0.001	0.436
	V1 vs. V10	0.24	-0.09	0.57			0.152	
	V1 vs. V12	0.99	0.56	1.41			< 0.001	
	V10 vs. V12	0.76	0.24	1.27			0.005	
Placebo	- V0V1V2V4V7V10V12				4.72	6, 23.16	0.003	0.209
	V1 vs. V10	0.20	-0.12	0.51			0.205	
	V1 vs. V12	0.62	0.23	1.01			0.004	
	V10 vs. V12	0.42	-0.06	0.90			0.003	
**PANSS negative** &								
Spiro 100 mg	- V0V1V2V4V7V10V12				2.08	6, 25.79	0.090	
Spiro 200 mg	- V0V1V2V4V7V10V12				4.20	6, 31.00	0.003	0.222
	V1 vs. V10	1.89	0.55	3.23			0.007	
	V1 vs. V12	2.52	0.33	4.70			0.026	
	V10 vs. V12	0.62	-1.87	3.12			0.614	
Placebo	- V0V1V2V4V7V10V12				1.68	6, 25.98	0.167	
**PANSS general** &								
Spiro 100 mg	- V0V1V2V4V7V10V12				2.54	6, 48.61	0.032	0.222
	V1 vs. V10	2.79	0.39	5.19			0.024	
	V1 vs. V12	n.d.	n.d.	n.d.			1.00	
	V10 vs. V12	n.d.	n.d.	n.d.			1.00	
Spiro 200 mg	- V0V1V2V4V7V10V12				2.59	6, 60.14	0.038	0.199
	V1 vs. V10	1.02	-0.49	2.52			0.176	
	V1 vs. V12	2.98	0.46	5.50			0.023	
	V10 vs. V12	1.96	-0.90	4.83			0.172	
Placebo	- V0V1V2V4V7V10V12				3.29	6, 21.88	0.018	0.153
	V1 vs. V10	0.59	-1.75	2.94			0.603	
	V1 vs. V12	2.93	-0.73	6.59			0.108	
	V10 vs. V12	2.33	-1.86	6.53			0.263	
**PANSS total** &								
Spiro 100 mg	- V0V1V2V4V7V10V12				2.92	6, 23.01	0.029	0.204
	V1 vs. V10	5.89	1.54	10.26			0.010	
	V1 vs. V12	6.80	0.50	13.10			0.036	
	V10 vs. V12	0.90	-6.40	8.21			0.802	
Spiro 200 mg	- V0V1V2V4V7V10V12				6.62	6, 27.23	< 0.001	0.257
	V1 vs. V10	3.63	0.42	6.85			0.028	
	V1 vs. V12	8.48	3.82	13.13			0.001	
	V10 vs. V12	4.85	-0.65	10.34			0.082	
Placebo	- V0V1V2V4V7V10V12				3.44	6. 23.17	0.014	0.166
	V1 vs. V10	1.63	-2.38	5.65			0.408	
	V1 vs. V12	6.52	1.25	11.87			0.019	
	V10 vs. V12	4.93	-1.45	11.31			0.125	

Supplementary Table 7: Post hoc time effects separately for groups (PANSS data): * based on Rankit transformed variables; # based on estimated marginal means; post hoc analysis between timepoints listed only, if main effects for the respective group was significant; & not all post hoc analysis between timepoints listed; n.d.: not defined

Supplementary Note 9

Further secondary outcomes

We used the Positive and Negative Syndrome Scale (PANSS) to measure psychopathology in our schizophrenia patients at several timepoints of the study. PANSS is a 30-item rating scale that has been developed to investigate the severity of psychopathology in schizophrenia patients. The scale is composed of three subscales (positive, negative, general) and one total scale. As PANSS was assessed more frequently during the study course than the aforementioned secondary endpoints, PANSS outcomes are presented separately.

Please see Supplementary **Figure 1** for the course of the PANSS subscales and the visualisation of the Rankit transformed data. For all PANSS analyses no significant time x group interaction could be observed in the LMM analyses:

- **PANSS$_{\textbf{Positive}}$** $F_{(12, 72.495)}=1.100$, $p=0.374$
- **PANSS$_{\textbf{Negative}}$** $F_{(12, 71.851)}=1.015$, $p=0.445$
- **PANSS$_{\textbf{General}}$** $F_{(12, 67.873)}=1.097$, $p=0.377$
- **PANSS$_{\textbf{Total}}$** $F_{(12, 72.261)}=1.518$, $p=0.138$

In general, a subtle decrease in all PANSS values over the trial period was observed. This pattern did not show any differences across groups and can be expected in such trials as being in a trial provides social and emotional support for study patients by the research teams resulting in a secondary improvement of psychopathology.

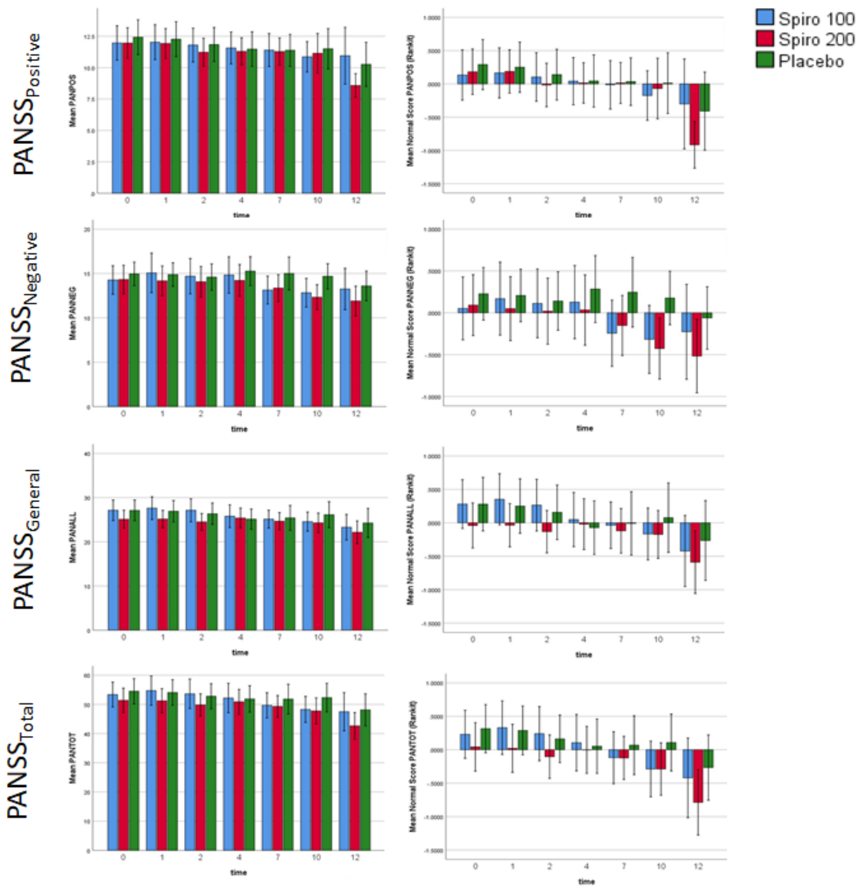

Supplementary Figure 1: Visualisation of PANSS outcome data (left: mean values; right: Rankit transformed data). Error bars refer to 95%CI

Supplementary Note 10

Supplementary laboratory measures (potassium, sodium, creatinine)

As electrolyte abnormalities are known and an effect of spironolactone was expected, special attention was paid to the courses of sodium (Na) and potassium (KAL) and the creatinine (KREA) values. As expected, we were able to observe an increase in potassium levels and a decrease in sodium levels during the intervention period that normalized after the end of the intervention. These analyses are presented for all available timepoints. LMM showed a significant time x group interaction (p=0.005) and several other expected effects for the potassium (KAL) level analyses. **Supplementary Figure 2** shows the course of potassium levels over time (visit 1 to 12) with raw and Rankit transformed values. Please see **Supplementary Table 1** for the respective analyses.

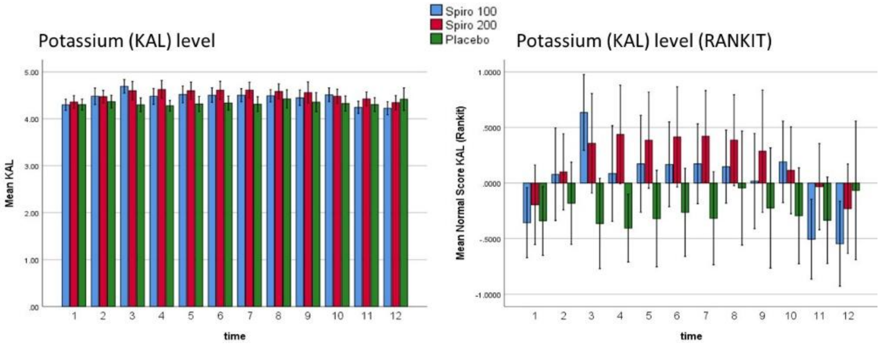

Supplementary Figure 2: Visualisation of potassium levels data (left: mean values; right: Rankit transformed data). Error bars refer to 95%CI

Type III Tests of Fixed Effects[a]

Source	Numerator df	Denominator df	F	Sig.
Intercept	1	68.076	0.786	0.378
Time	11	68.208	4.955	0.000
Gruppe	2	71.832	4.795	0.011
Gender	1	72.833	2.418	0.124
Center	2	65.563	7.764	0.001
Age	1	68.418	4.789	0.032
School_Years	1	66.539	0.145	0.704
Time * Gruppe	22	69.712	2.256	0.005

Supplementary Table 5: LMM outcome for the potassium analyses (performed on Rankit transformed data)

Despite Rankit transformation, the assumption of normality was not met for the sodium analyses. Thus, non-parametric tests were used. Kruskal-Wallis tests showed significant between-group differences for visits V4 (p=0.003), V5 (p=0.002), V6 (p=0.005), V7 (p=0.003) and V8 (p=0.015). All other visits showed no between-group differences (all p > 0.098). In all visits with significant between-group differences the spironolactone groups showed significant differences or were on trend level in the Sidak-corrected Mann-Whitney U-tests compared to placebo (all p between 0.056 and 0.003), but not between each other (all p > 0.686). Please see **Supplementary Figure 3** for the visualization of the sodium level course and the Rankit transformed data for the visualisation of between group differences.

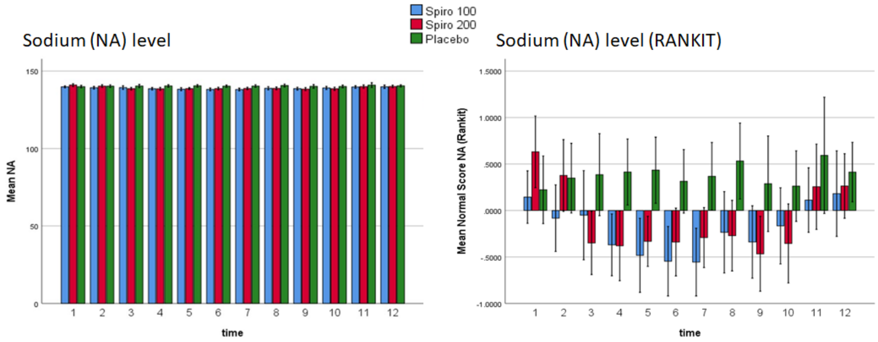

Supplementary Figure 3: Visualisation of sodium data (left: mean values; right: Rankit transformed data). Error bars refer to 95%CI

Despite Rankit transformation, the assumption of normality was not met for the creatinine analyses. Thus, non-parametric tests were used. Kruskal-Wallis tests showed significant between-group differences for visits V5 (p=0.042) and V9 (p=0.039). All other visits showed non-significant between-group differences (all p > 0.056). In visit V5 a significant difference in creatinine between the spironolactone 200mg and the placebo group (p=0.036) was shown using Sidak-corrected Mann-Whitney U-Tests. In visit V9 the same pattern could be observed (p=0.033). Please see **Supplementary Figure 4** for the visualization of the sodium level course and the Rankit transformed data for the visualisation of between-group differences.

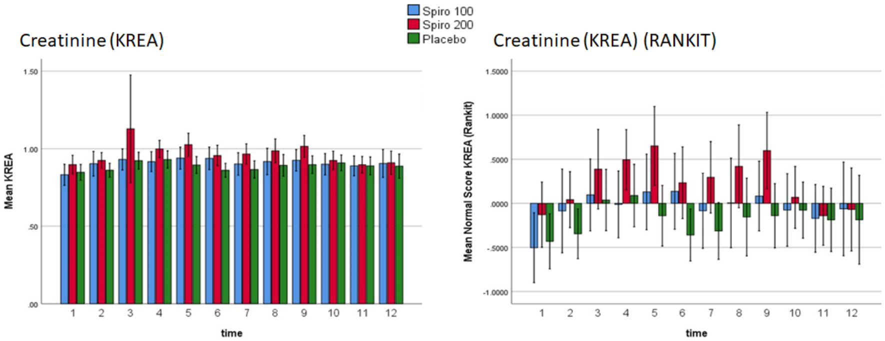

Supplementary Figure 4: Visualisation of creatinine data (left: mean values; right: Rankit transformed data). Error bars refer to 95%CI

Supplementary Note 11

Blood pressure and heart rate

Including data of all points in time LMM did not show significant time x group interactions for systolic ($F_{(14,\ 70.847)}$=0.512, p=0.919) and diastolic blood pressure values ($F_{(14,\ 70.444)}$=0.756, p=0.712). However, for systolic blood pressure a significant (p=0.027) and for diastolic blood pressure a trend (p=0.052) was observed for within subject-fator time. This can be explained by the expected effect of a blood pressure-lowering effect of spironolactone during the intervention period. Please see **Supplementary igures 5 and 6** for a presentation of the course of blood pressure (BP).

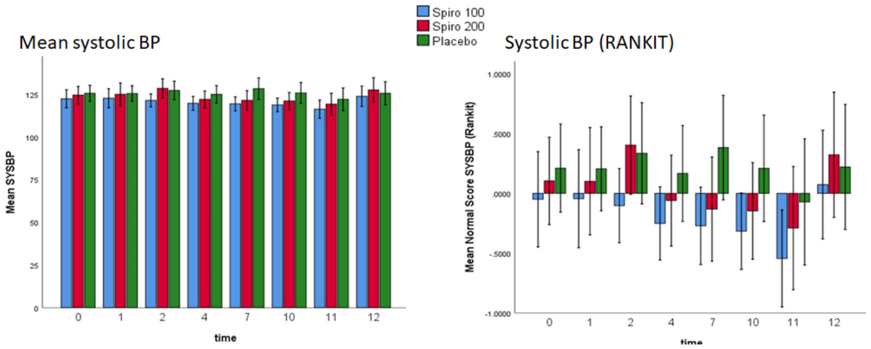

Supplementary Figure 5: Visualisation of systolic blood pressure (BP) data (left: mean values; right: Rankit transformed data). Error bars refer to 95%CI

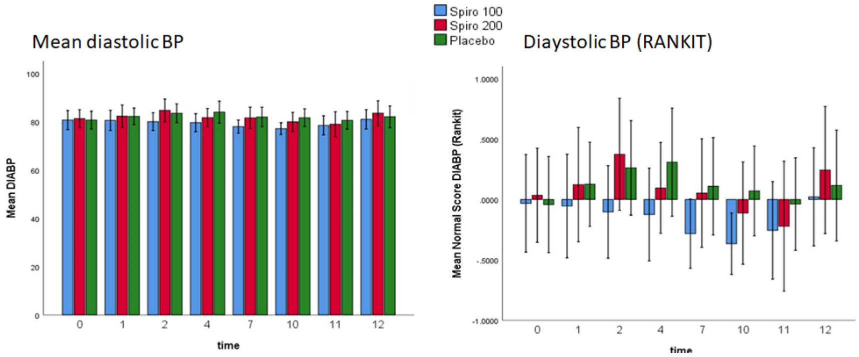

Supplementary Figure 6: Visualisation of diasystolic blood pressure (BP) data (left: mean values; right: Rankit transformed data). Error bars refer to 95%CI

For heart rate, no significant time x group interaction (p=0.713) and no significant effect of time (0.071) was observed. Please see **Supplementary Figure 6** for a detailed presentation of the course of heart rate values over time.

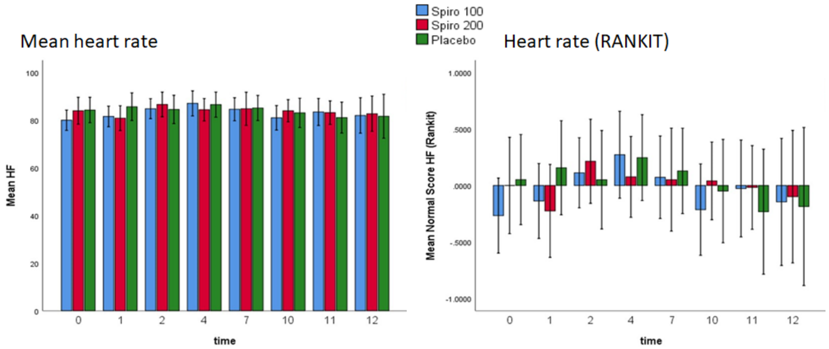

Supplementary Figure 6: Visualisation of heart rate data (left: mean values; right: Rankit transformed data). Error bars refer to 95%CI

Supplementary Note 12

Supplementary safety measures

Body mass index (BMI)

For BMI, no significant time x group interaction (p=0.540) and no significant effect of time (0.143) was observed. Please see **Supplementary Figure 7** for a presentation of the course of BMI.

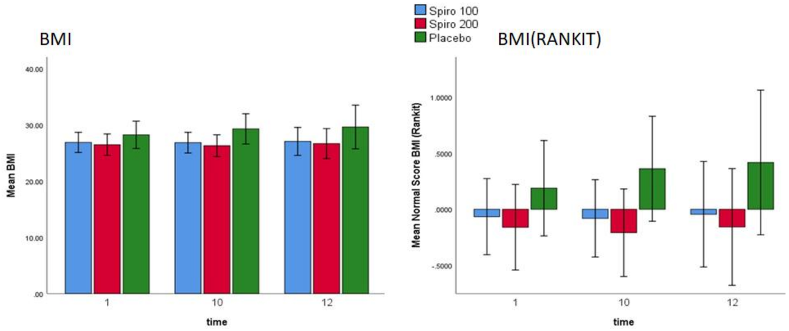

Supplementary Figure 6: Visualisation of BMI data (left: mean values; right: Rankit transformed data). Error bars refer to 95%CI

Motor side-effect

For the Simpson-Angus scale (SiAS), Kruskal-Wallis tests revealed no significant group differences for V1, V10 or V12 (all p > 0.214).

Supplementary Note 13

Supplementary information regarding AE/SAE

Adverse Events and Serious Adverse Events were classified according to CTCAE V. 4.0 and coded according to MedDRA V. 23.1 (English). A total of 154 AEs were reported in 84 participants. In the ITT sample 152 AEs appeared. While not reaching significance (p=0.088), numeric distribution indicates that in both study groups more AEs occurred with no significant differences across these two groups compared to the placebo group. 121 AE were rated Grade 1 (mild), 27 Grade 2 (moderate), 4 Grade 3 (severe), 0 Grade 4 (life-threatening), 0 Grade 5 (death). No significant differences could be observed across all three study groups (p = 0.164)

Supplementary Table 8: Cumulative Summary Tabulation of Adverse Events

**MedDRA System Organ Class. SOC** Preferred Term. PT	**Results (N)**	**%**
**General disorders and administration site conditions**	**16**	**10.39**
Asthenia	1	
Thirst	1	
Fatigue	10	
Flu-like illness	1	
Edema peripheral	1	
Pain	1	
Lethargy	1	
**Eye disorders**	**1**	**0.65**
Blepharospasm	1	
**Surgical and medical procedures**	**2**	**1.3**
Hospitalization	1	
Varicose vein operation	1	
**Reproductive system and breast disorders**	**4**	**2.6**
Amenorrhea	2	
Dysmenorrhea	1	
Testicular pain	1	
**Skin and subcutaneous tissue disorders**	**3**	**1.95**
Rash	1	
Eczema	1	
Hyperhidrosis	1	
**Renal und urinary disorders**	**10**	**6.49**
Urge incontinence	2	
Dysuria	1	
Urgency urination	2	
Kidney pain	1	
Pollakisuria	4	
**Blood and lymphatic system disorders**	**5**	**3.25**
Leukocytosis	3	
Lymphadenopathy	1	
Thrombocytosis	1	
**Gastrointestinal disorders**	**11**	**7.14**
Abdominal tenderness	1	
Abdominal pain	2	
Diarrhea	2	
Dyspepsia	1	
Gastroesophageal reflux disease	1	
Hypersalivation	1	
Obstipation	1	
Nausea	2	
**Nervous system disorders**	**29**	**18.83**
Akathisia	1	
Dysmetria	1	

MedDRA System Organ Class. SOC preferred term. pt	Results	%
Headache	5	
Psychomotor hyperactivity	1	
Orthostatic dizziness	2	
Dizziness	11	
Dizziness exertional	1	
Somnolence	1	
Restless leg syndrome	1	
Tremor	5	
**Vascular disorders**	**4**	**2.6**
Hypertension	1	
Hypotension	1	
Peripheral venous disease	1	
Varicose veins of lower extremities	1	
**Cardiac disorders**	**8**	**5.19**
Arrhythmia	1	
Bradykardia	1	
Palpitations	1	
Supraventricular extrasystoles	1	
Tachykardia	3	
Ventricular extrasystoles	1	
**Infections and infestations**	**10**	**6.49**
Bacterial infection	1	
Febrile infection	1	
Infection	1	
Upper respiratory tract infection	1	
Nasopharyngitis	3	
Picornavirus infection	1	
Subcutaneous abscess	1	
Viral infection	1	
**Psychiatric disoders**	**23**	**14.94**
Affective disorder	1	
Fear	1	
Apathy	3	
Depression	1	
Affect lack	1	
Hallucinations	1	
Psychosis	6	
Insomnia	1	
Sleep disorder	3	
Suicidal ideation	1	
Restlessness	4	
**Musculoskeletal and connective tissue disorders**	**6**	**3.9**
Arthralgia	1	
Muscle spasms	2	
**MedDRA System Organ Class. SOC preferred term. pt**	**Results**	**%**
Musculoskeletal stiffness	2	
Musculoskeletal pain	1	
**Metabolism and nutrition disorders**	**16**	**10.39**
Decreased appetite	2	
Folate dificiency	1	
Hyperkalemia	13	
**Investigations**	**6**	**3.9**
C-reactive protein increased	1	
Weight increased	1	
Blood potassium increased	3	
Blood creatinine increased	1	
**Overall Result**	**154**	**100**

Two SAEs were observed in two different patients (one female (age range 40 to 50), one male (age range 30 to 40)) during the conduction of the trial. Both were re-hospitalization due to to worsening of psychotic symptoms and occurred at the LMU site. Both SAEs were rated as moderate and not related to the study treatment and the outcome was recovery from the SAE. One SAE occurred in the placebo group and one SAE occurred in the spironolactone 200 mg group. No Suspected Unexpected Serious Adverse Reactions (SUSAR) were reported in the study

A detailed overview of Serious Adverse Events and more data regarding AEs and SAEs can be requested from the authors. Please sent you request to the first author alkomiet.hasan@med.uni-augsburg.de and to the CRO (muenchner.studienzentrum@mri.tum.de). All requests will be verified within four weeks after first request and data will be provided after approval of the trial statistician (TSA) and responsible PIs (AH, PF).
