# Supplementary material for: Antagonizing NRG1-ERBB4 signaling pathway with spironolactone for the treatment of schizophrenia: results of a randomized controlled drug repositioning clinical trial
Source: Commun Med (Lond). 2026 Jun 16;6:337. doi: 10.1038/s43856-026-01714-3 (PMC13272664; doi:10.1038/s43856-026-01714-3)
Supplement: Supplementary file 3 — Description of Additional Supplementary Files [file 43856_2026_1714_MOESM3_ESM.pdf]

Description of Additional Supplementary Files

File name: Supplementary Data 1

Description: Anonymized individual patient data to understand the main outcome analyses presented in this publication

File name: Supplementary Data 2

Description: All values for the development of the figures
