# Supplementary material for: Antagonizing NRG1-ERBB4 signaling pathway with spironolactone for the treatment of schizophrenia: results of a randomized controlled drug repositioning clinical trial
Source: Commun Med (Lond). 2026 Jun 16;6:337. doi: 10.1038/s43856-026-01714-3 (PMC13272664; doi:10.1038/s43856-026-01714-3)
Supplement: Supplementary file 1 — Transparent Peer Review file [file 43856_2026_1714_MOESM1_ESM.pdf]

nature portfolio

Peer Review File

Antagonizing NRG1-ERBB4 signaling pathway with spironolactone for the treatment of schizophrenia: results of a randomized controlled drug repositioning clinical trial

Corresponding Author: Professor Alkomiet Hasan

This file contains all reviewer reports in order by version, followed by all author rebuttals in order by version.

Version 0:

Reviewer comments:

Reviewer #1

(Remarks to the Author)
This manuscript provides a well-detailed summary of the proposed mechanism of action of this potential therapeutic option and the clinical study design. The methods appear appropriate and replicable. I have no concerns or suggestions for improvement.

Reviewer #2

(Remarks to the Author)
Thank you for the opportunity to review a very interesting paper. Your paper is concise, well written and the description of the statistical modelling procedure in the paper and the SAP are exemplary.

1. As I understand it, when looking at the timepoints (v1 -> v10) there was no significant treatment time interaction, with the same finding when including v12 in the model. There was however a significant effect for time alone. A post-hoc analysis between visits did find significant results for both intervention groups. If I have misunderstood this then please do make this clearer in the results - Primary outcome measure section (lines 124-136).

2. If there is a particular reason why the post-hoc analysis was chosen to be a binomial model, then please make this clear.

3. As far as I can see there is no mention of what your primary outcome is until it is mentioned in the results section. As the methods will be only online it may be good to add a line into the introduction briefly explaining the primary outcome, as this would help readers make sense of the 1 and 0 hit secondary outcomes.

4. In your CONSORT diagram, if you are aware of the reasons for loss to follow up, please enter them (something like uncontactable is appropriate if the participant disappeared). Otherwise, please remove the (give reasons) from the follow-up boxes.

5. In relation to table 1, could you explain why you have decided to test for differences between arms? By randomising participants to the treatment these tests would all be expected to give no significant difference as randomisation should balance scores and characteristics at baseline, especially in a reasonable sample size like the one the study has.

I look forward to seeing your responses.
Callum Glen
Trial Statistician, King's College, London

Version 1:

Reviewer comments:

Reviewer #2

(Remarks to the Author)
Thank you for your revisions. I have no concerns or suggestions on the manuscript in its current form.

Open Access This Peer Review File is licensed under a Creative Commons Attribution 4.0 International License, which permits use, sharing, adaptation, distribution and reproduction in any medium or format, as long as you give appropriate credit to the original author(s) and the source, provide a link to the Creative Commons license, and indicate if changes were made.
In cases where reviewers are anonymous, credit should be given to 'Anonymous Referee' and the source.
The images or other third party material in this Peer Review File are included in the article's Creative Commons license, unless indicated otherwise in a credit line to the material. If material is not included in the article's Creative Commons license and your intended use is not permitted by statutory regulation or exceeds the permitted use, you will need to obtain permission directly from the copyright holder.
To view a copy of this license, visit https://creativecommons.org/licenses/by/4.0/

Universität Augsburg
Medizinische Fakultät

Universität Augsburg – Klinik für Psychiatrie, Psychotherapie und Psychosomatik –
Geschwister-Schönert Straße 1 – 86135 Augsburg

Univ. Prof. Dr. Alkomiet Hasan

Direktor
Klinik für Psychiatrie, Psychotherapie und Psychosomatik der Universität Augsburg

Vorstand Krankenversorgung
Bezirkskliniken Schwaben

Prodekan
Medizinische Fakultät

Geschwister-Schönert Straße 1
86156 Augsburg
Telefon +49 (0) 821 4803 - 1001
Telefax +49 (0) 821 4803 - 1002
alkomiet.hasan@med.uni-augsburg.de

Augsburg, 13th April 2026

COMMSMED-25-3207-T

Antagonizing NRG1-ERBB4 signaling pathway with spironolactone for the treatment of schizophrenia: results of a randomized controlled drug repositioning clinical trial

Thank you very much for giving us the possibility to revise and resubmit our manuscript. Please find attached our responses to the reviewer's comments.

Reviewer #1

Comment: This manuscript provides a well-detailed summary of the proposed mechanism of action of this potential therapeutic option and the clinical study design. The methods appear appropriate and replicable. I have no concerns or suggestions for improvement.

Response: Thank you very much for your positive evaluation and your support.

Reviewer #2

Comment: Thank you for the opportunity to review a very interesting paper. Your paper is concise, well written and the description of the statistical modelling procedure in the paper and the SAP are exemplary.

Response: Thank you very much for your very positive evaluation.

Comment 1: As I understand it, when looking at the timepoints (v1 → v10) there was no significant treatment time interaction, with the same finding when including v12 in the model. There was however a significant effect for time alone. A post-hoc analysis between visits did find significant results for both intervention groups. If I have misunderstood this then please do make this clearer in the results - Primary outcome measure section (lines 124-136).

Response 1: Thank you very much for your comment. We observed no significant treatment x time interaction, but for the primary outcome measure we observed significant effects for factor time for both intervention groups (spironolactone 100mg: $p = 0.017$, V1 vs. V10: $p = 0.026$, V1 vs. V12: $p = 0.022$, V10 vs. V12: n.s., spironolactone 200mg: $p = 0.004$, V1 vs. V10: $p = 0.011$, V1 vs. V12: $p < 0.001$, V10 vs. V12: n.s.). We observed this pattern of results also for several secondary outcome measures. Unfortunately, in the original submission Supplementary results table 4 that summarizes the post hoc comparisons between

Universität Augsburg University

visits V1, V10, V12 was missing. We would like to apologize for this unnecessary error. We now have inserted this table that illustrates the time effects in both intervention groups in the Supplementary Material. Additionally, we now present the results in the primary outcome section more detailed. Finally, we have revised the supplementary material to make it more reader friendly.

Comment 2: If there is a particular reason why the post-hoc analysis was chosen to be a binomial model, then please make this clear.

Response 2: We thank the reviewer for this important question. The primary analysis specified in the SAP was a linear mixed model (LMM) on relative hit rates, which represents the pre-specified and confirmatory analysis for this trial. The binomial GLMM was added as an unplanned exploratory sensitivity analysis to complement the primary analysis. This model allows the underlying count structure of the data (number of correct hits out of total trials) to be appropriately modelled while accounting for within-subject correlation. However, as this analysis was not pre-specified, it should be interpreted as exploratory and supportive in nature. We revised figure 5 and now all information between text and figure correspond.

Comment 3: As far as I can see there is no mention of what your primary outcome is until it is mentioned in the results section. As the methods will be only online it may be good to add a line into the introduction briefly explaining the primary outcome, as this would help readers make sense of the 1 and 0 hit secondary outcomes.

Response 3: For the revision, we had to re-arrange the order of sections. Now, the primary outcome is described as part of the methods section before presenting the results.

Comment 4: In your CONSORT diagram, if you are aware of the reasons for loss to follow up, please enter them (something like uncontactable is appropriate if the participant disappeared). Otherwise, please remove the (give reasons) from the follow-up boxes.

Response 4: Thank you very much for bringing this to our attention. In the module End of Study from the trial CRF we have data on lost to follow-up (yes/no). If lost to follow-up was indicated, there was unfortunately no further information. We removed “(give reason)” from the boxes.

Comment 5: In relation to table 1, could you explain why you have decided to test for differences between arms? By randomising participants to the treatment these tests would all be expected to give no significant difference as randomisation should balance scores and characteristics at baseline, especially in a reasonable sample size like the one the study has.

Response 5: We thank the reviewer for your comment. We agree that formal statistical testing of baseline characteristics is not required in randomized trials, as randomization is expected to ensure comparability between groups. These tests were included for descriptive purposes only and do not affect the interpretation of the results. We have clarified this in the manuscript.

Yours sincerely, also on behalf of the co-authors,

Alkomiet Hasan
